# Genome-wide identification of nucleotide-binding domain leucine-rich repeat (NLR) genes and their association with green peach aphid (*Myzus persicae*) resistance in peach

**DOI:** 10.1186/s12870-023-04474-7

**Published:** 2023-10-25

**Authors:** Haixiang Yu, Xuelian Wu, Jiahui Liang, Ziying Han, Yuansong Xiao, Hao Du, Yihua Liu, Jian Guo, Futian Peng

**Affiliations:** 1 College of Horticulture Science and Engineering, Shandong Agricultural University, Tai’an, Shandong China; 2 College of Agriculture and Forestry Sciences, Linyi University, Linyi, Shandong 276000 China

**Keywords:** Peach, Aphid, Nucleotide-binding domain leucine-rich repeat (NLR) genes, Genome-wide analysis

## Abstract

Resistance genes (R genes) are a class of genes that are immune to a wide range of diseases and pests. In planta, NLR genes are essential components of the innate immune system. Currently, genes belonging to NLR family have been found in a number of plant species, but little is known in peach. Here, 286 NLR genes were identified on peach genome by using their homologous genes in *Arabidopsis thaliana* as queries. These 286 NLR genes contained at least one NBS domain and LRR domain. Phylogenetic and N-terminal domain analysis showed that these NLRs could be separated into four subfamilies (I-IV) and their promoters contained many cis-elements in response to defense and phytohormones. In addition, transcriptome analysis showed that 22 NLR genes were up-regulated after infected by Green Peach Aphid (GPA), and showed different expression patterns. This study clarified the NLR gene family and their potential functions in aphid resistance process. The candidate NLR genes might be useful in illustrating the mechanism of aphid resistance in peach.

**Supplementary Information:**

The online version contains supplementary material available at 10.1186/s12870-023-04474-7.

## Introduction

The plant innate immunity system ensures normal growth during pathogen infection [1]. Plants have evolved cell surface and intracellular receptors that can recognize pathogen-derived chemicals or molecules. There were two layers of immune system, called pattern-triggered immunity (PTI) and effector triggered immunity (ETI) [1]. PTI is induced when surface pattern-recognition receptors (PRRs) bind pathogen-derived molecules at the plasma membrane. ETI generally is induced inside cells, when pathogen virulence factors (known as effectors) are recognized by NLR receptors, thereby inducing immune responses [2–4].

NLR genes are the most important R genes in plants [5]. The proteins encoded by these genes are highly similar and usually have three conserved domains: the NBS domain with the core in the middle, the LRR domain with different numbers at the C-terminal and the variable domain at the N-terminal [6]. In angiosperms, NLR genes were mainly characterized into two categories: TIR-NBS-LRR (TNL) and non-TIR-NBS-LRR (nTNL), which is also known as CC-NBS-LRR (CNL) [6]. Recently, some other types of domains were identified, such as resistance to powdery mildew (RPW8) domain, which consist of Transmembrane-Coiled-coiled (TM-CC) domain. [7]. It was reported that the NLR gene encoding the TM-CC domain was not directly involved in the recognition of specific pathogens, but participated in downstream signaling pathway of disease resistance process. For example, *NRG1* (*DQ054580.1*) in tobacco and *ADR1* (*AT1G33560.1*) in *Arabidopsis thaliana* can both regulate the accumulation of the defense hormone salicylic acid during the immune response, and *ADR1* can also be used as “auxiliary NBS-LRR” to transduce specific NBS-LRR receptors during ETI [6, 8]. P-kinase, Hydrolase and Duf676 are new domains found in the N-terminal of R protein, which were identified in the genomes of *Physcomitrella patens*, *Marchantia polymorpha*, and *sphagnum fallax* respectively, but the functions of these bryophyte-specific NLR subclasses have not yet been explored [9–11].

The NB-ARC structure (NBS) domain, belongs to the signal transducing ATPase multi-structural domain (STAND) superfamily [12], which has function in binding and hydrolyzing ATP [13, 14]. In *Arabidopsis thaliana*, it was identified that the NBS domain usually contains 8 conserved motifs [15], including P-loop, RNBS-A, kinase2, RNBS-B, RNBS-C, GLPL, RNBS-D and MHDV. These motifs are all conserved in the NBS domain of other species [16]. Kinase 2 may be an important regulator of ATP hydrolysis, and P-loop, GLPL and MHDV, which may be involved in the regulation of nucleotide binding. The mutation of aspartate in MHDV region of *tomato I-2* resulted in continuous activation [17]. In the P-loop region of *RPM1* and other NLR genes in *Arabidopsis thaliana* showed that the proteins were inactivated [18]. The leucine rich repeat (LRR) domain is more polymorphic than the NBS domain, which is composed of 20–30 leucine rich residues and forms β chain α Spiral structure [19]. Therefore, LRR domains are often involved in protein-protein interactions. Some pathogens, including *Listeria* and *Streptococcus*, can integrate into host cells by encoding proteins with LRR domains [20].

Plants rely on NLR protein to respond to invasive pathogens and activate the immune response, so as to obtain resistance to bacteria, viruses, nematodes and pests [21]. In previous studies, many NLR proteins that are resistant to pests have been proved in different plants. For example, *Rpi-blb2* confer broad-spectrum resistance to pathogen isolates in potato [22]. *Mi-1.2* is similar to *Rpi-blb2*, it has specific resistance to root knot nematodes and aphids in tomato [23]. In gramineous plants, the resistance of wheat to aphids was dominated by *Adnr1* [24]. The *RMES1* locus which contains five NLR genes on sorghum genome were predicted, and proven resistance to *Melanaphis sacchari* [25]. In addition, the *Dp-fl* locus, which confers resistance to *Dysaphis plantaginea* contains 19 genes acting as R-genes, 2 of which are NLRs in *Malus pumila* [26].

Peach is the fourth largest deciduous fruit crop in the world and has valuable nutrition [27]. Green Peach Aphid (*Myzus persicae*, GPA) is the most harmful pest during peach production. It can stab and suck the new shoots and leaves, resulting in curling leaves, growth limitations. It can also secrete honeydew to spread viruses between species. In the last decades, several genetic loci conferring resistance to aphids have been identified and mapped on peach genome. Most of genes belong to the resistance genes encoding NLR proteins [28]. In peach, a strong candidate gene responsible for the dominant GPA resistance in *Rm3* locus were identified [29]. However, the regulatory mechanism and other NLR genes in response to GPA infestation were still unknown.

In this study, we analyzed the NLR gene family in peach. A total of 286 NLR genes were identified, and their chromosome location, phylogenetic relationship, gene structure, conserved domains and promoter cis-elements were analyzed. Transcriptome analysis showed that the expression of 22 identified NLR genes was significantly up-regulated after GPA infestation. The results would provide a basis for further study on the function of NLR genes in aphid resistance.

## Result

### Identification and distribution of NLR gene family in peach

**Fig. 1:**
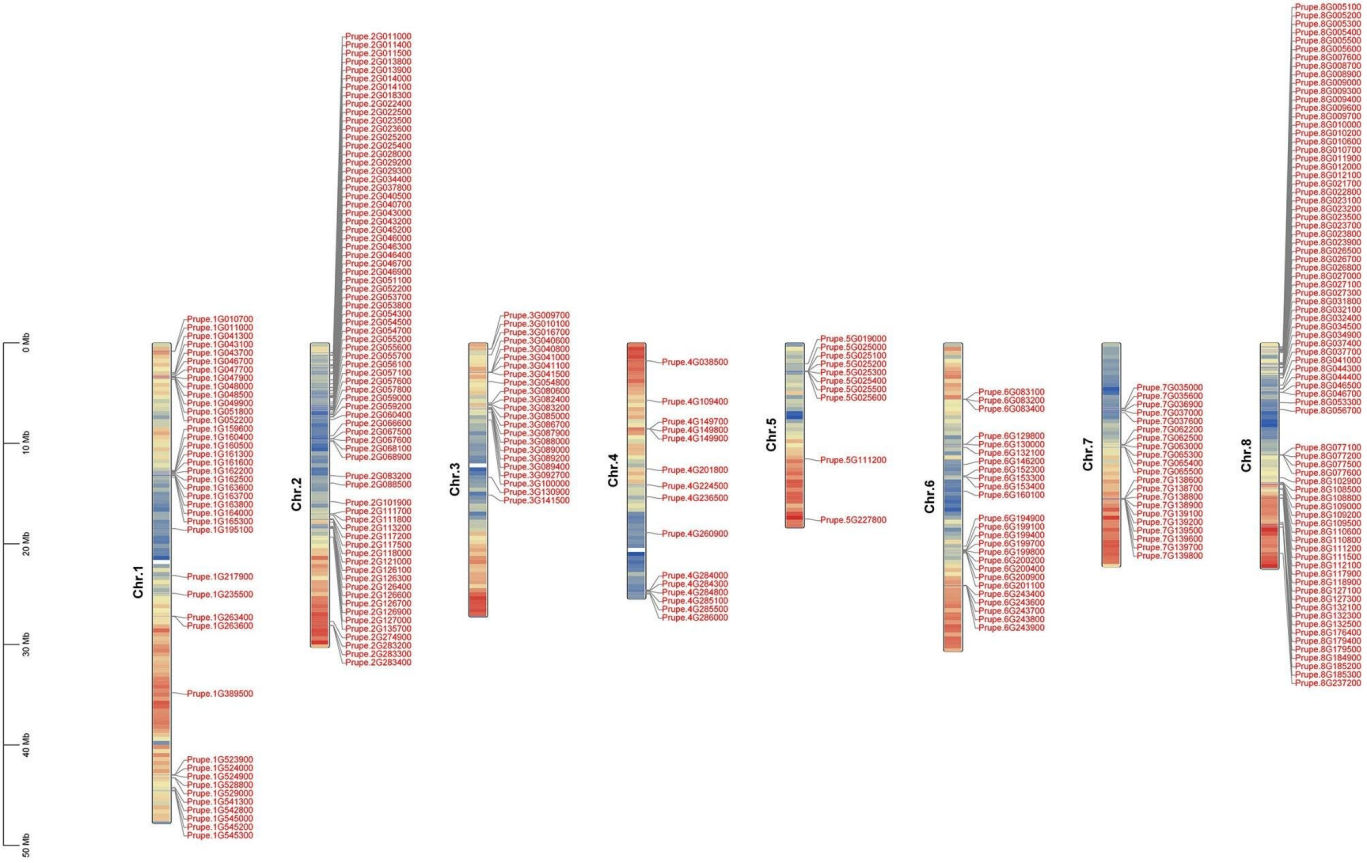
Distribution of peach NLR gene family on peach genome. Chromosomes 1–8 are indicated by bars of gene density, and peach NLR genes are marked in red font

The NLR genes in peach genome were identified according to the NBS and LRR domain. Firstly, the 195 NLR genes in *Arabidopsis thaliana* were used as queries to find out the candidate genes in peach using the NCBI-Blastp toolkit. Then, their protein domains were further analyzed, especially the number of NBS and LRR domain. Finally, 286 NLR genes were selected in this study, which showed at least one NBS and LRR domain. These NLR genes are unevenly distributed on peach chromosomes, most of which are present on chr.1 (14.3%), chr.2 (25.52%) and chr.8 (27.27%) (Fig. 1).

The length of amino acids for these NLR proteins ranged from 421 (*Prupe.4G236500*) to 2026 (*Prupe.7G065400*), with an average length of 1055. The molecular weight ranged from 48041.58 Da (*Prupe.4G236500*) to 230015.7 Da (*Prupe.7G065400*), with an average of 120157.56 Da. The isoelectric point of these NLR proteins ranged from 5.14 (*Prupe.8G110800*) to 9.61 (*Prupe.3G040800*), with an average value of 6.99, indicating that peach NLRs are mostly neutral protein (Table S1).

### Phylogenetic relationships, domains, motifs and number of exons of peach NLR gene family

**Fig. 2:**
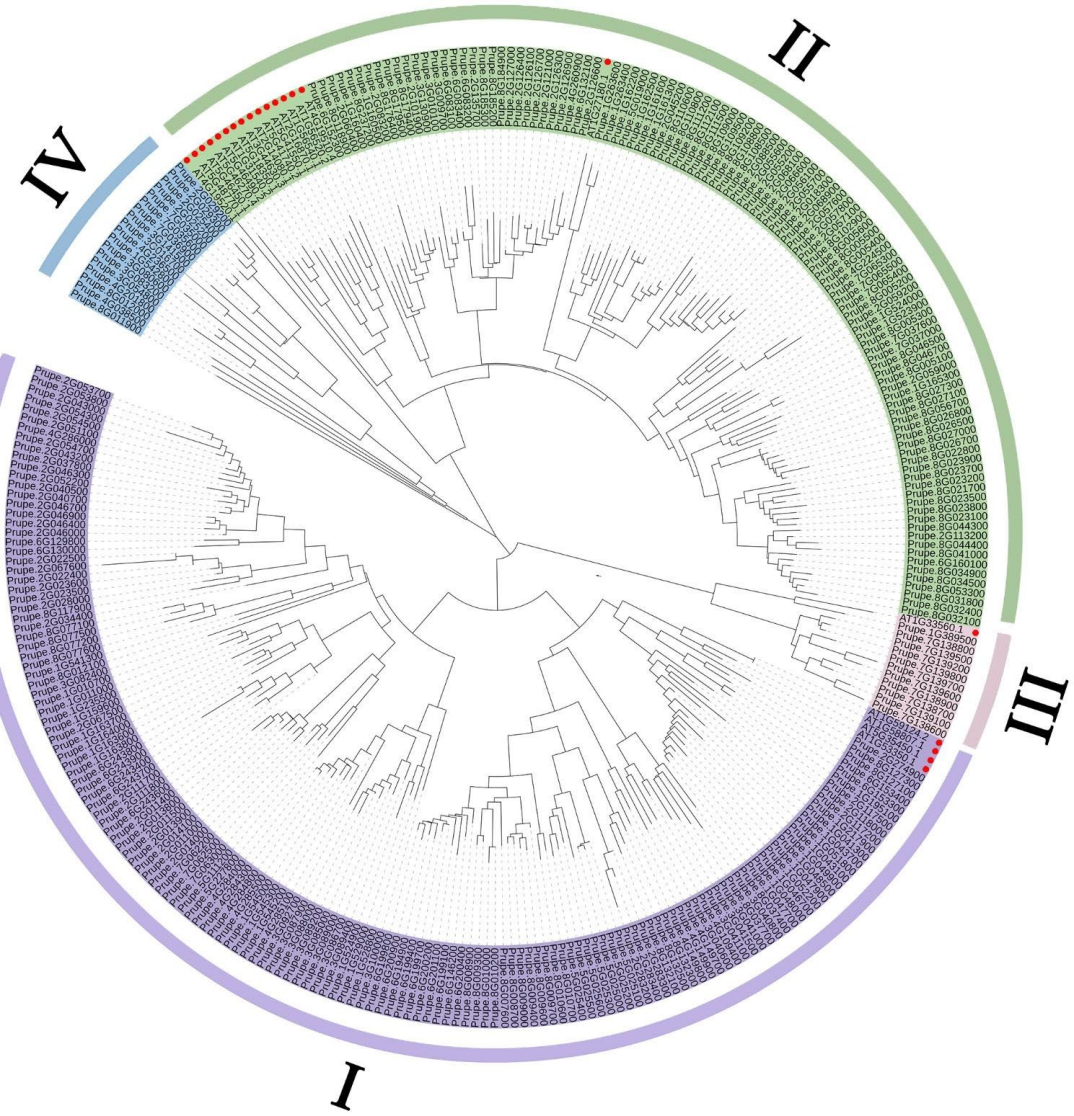
Phylogenetic analysis of peach NLR family. Subfamily I indicates CNL, Subfamily II indicates TNL and subfamily III indicates RNL. Subfamily IV contained NLRs without N-terminal domain. The red circle represents *Arabidopsis thaliana*

To uncover the evolutionary relationship of the peach NLR genes, a neighbor-joining (NJ) phylogenetic tree was constructed using protein sequences of peach NLRs and 20 reported NLR genes in *Arabidopsis thaliana*, including *AT1G31540.2, AT1G56520.2, AT1G56540.1, AT2G14080.1, AT2G16870.1, AT3G44630.3, AT4G19510.4, AT4G19520.1, AT5G18350.1, AT5G41550.1, AT5G41740.2, AT5G46270.4, AT5G46450.1, AT5G46490.2, AT5G48770.1, AT1G53350.1, AT1G58807.1, AT1G59124.2, AT5G35450.1, AT1G33560.1*. The results showed that the peach NLR genes could be divided into four subfamilies (I-IV), which included 153, 104, 11, 18 peach NLR genes respectively (Fig. 2).

According to the differences in N-terminal domain, the subfamilies I-III were mainly characterized as CNL, TNL and RNL respectively, although some NLRs without N-terminal domain were also clustered in subfamily I and II (Fig. S1). By further checking the whole sequences of these NLRs, we found that the N-terminal conservative domain was not completely deleted, resulting in incomplete CC or TIR domains (Fig. S2). The subfamily IV contained NLRs without N-terminal domain. Phylogenetic analysis suggested that CNL, TNL and RNL were all derived from subfamily IV, which was consistent with the previous study [15].

Gene structure analysis of NLR gene family showed that peach NLR genes contained many Exon and UTRs, and there were significant differences among different subfamilies. The average numbers of Exon and UTRs of these NLR genes was 4.69 and 4.47. Besides, the numbers in subfamily I (3.31, 5.80) was mostly less than II (6.16, 4.19) (Table S2), while multiple exons were identified in subfamilies II, III. In contrast, the gene coding sequence of subfamily I and IV was simpler than the others. In addition, the smallest gene (*Prupe.4G236500*) has 3 Exons and no UTRs, much simpler than the longest (*Prupe.2G118000*) (4 Exons and 3 UTRs). (Fig. S3).

### Gene duplication and collinearity analysis

**Fig. 3:**
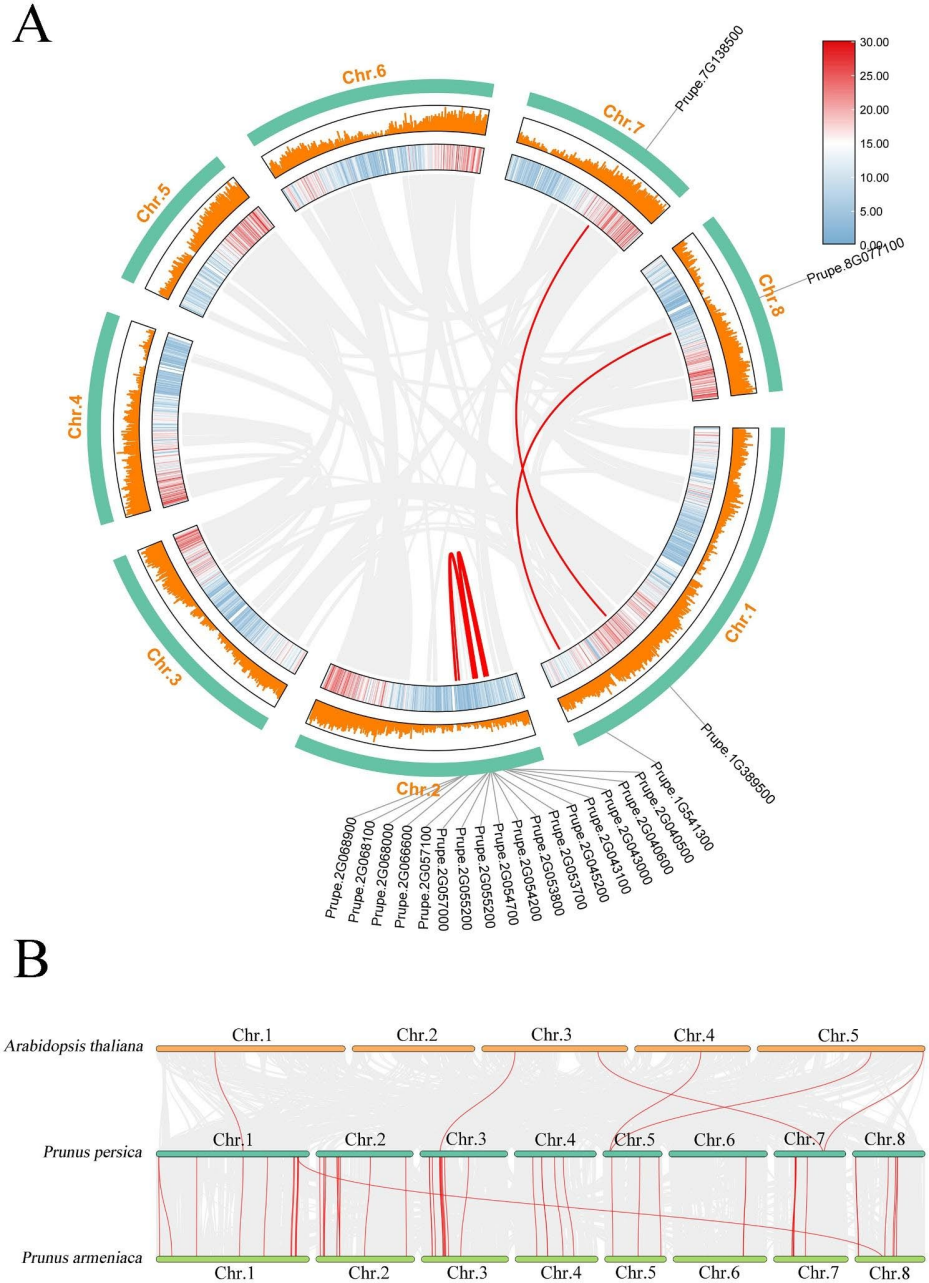
Collinearity analysis of NLR genes. The Chr means chromosomes, and the red lines represent the connections of collinear genes. **A** Collinearity analysis of peach NLR genes in peach. **B** NLR genes collinearity of *Prunus persica*, *Arabidopsis thaliana* and *Prunus armeniaca*

In order to further clarify the expansion and evolution of peach NLR genes and gene duplication events were investigated. Totally, 9 pairs of homologous gene on peach genome (*Prupe.1G389500/Prupe.7G138500, Prupe.1G541300/Prupe.8G077100, Prupe.2G055200/Prupe.2G066600, Prupe.2G057100/Prupe.2G068000, Prupe.2G040500/ Prupe.2G053700, Prupe.2G043000/ Prupe.2G504200, Prupe.2G045200/Prupe.2G055200, Prupe.2G055200/Prupe.2G068900, Prupe.2G057100/Prupe.2G068000*) were identified, indicating duplication was a major mode of gene expansion (Fig. 3A). In addition, we also constructed the collinearity of the NLR genes in the peach, *Arabidopsis thaliana* and *Prunus armeniaca*. A total of 6 pairs of NLR genes were identified between *Arabidopsis thaliana* and peaches, and 56 pairs of NLR genes also were identified between *Prunus armeniaca* and peach (Fig. 3B). This result showed that NLR genes in peach and *Arabidopsis thaliana* had relatively low homology, but high homology in *Prunus armeniaca* and peach. NLR copy number varies greatly across different species [30, 31]. Our results have shown that species with distant evolutionary relationships have much lower homology in NLR genes compared to plants of the same genus. Positive selection has been found in NLR genes, which contributed to make the NLR family is one of the most variable gene families in plant genomes [32, 33].

### Subcellular localization of peach NLRs

**Fig. 4:**
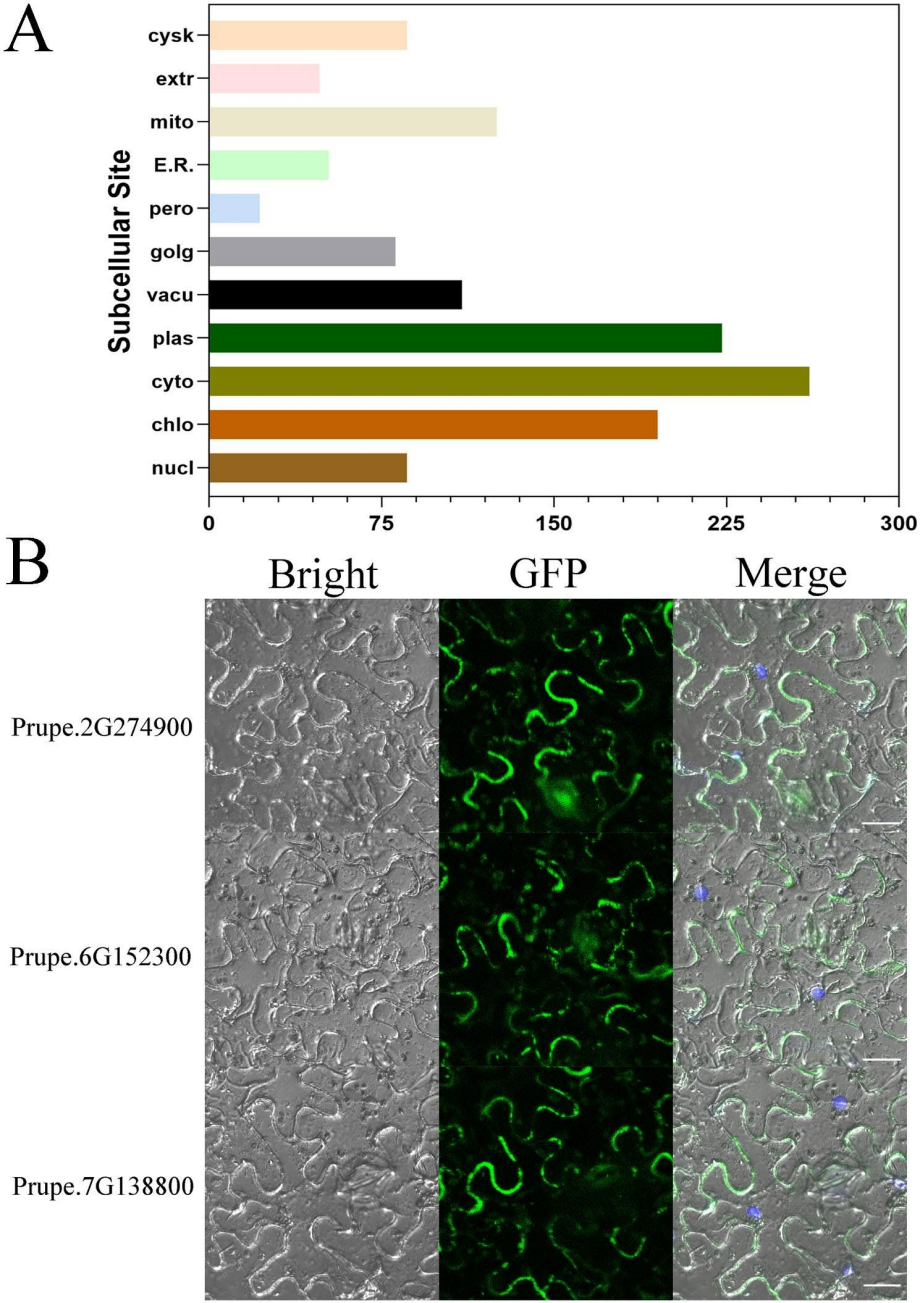
Subcellular localization of peach NLR genes. **A** Prediction of subcellular localization in wlof PSORT and Cello database. **B** Subcellular localization of three typical peach NLR proteins. The photographs were taken under bright light, in the dark feld for the GFP-derived green fourescence and merged, respectively. Scale bars, 20 μm

NLR has been shown to be expressed in the nucleus during effector induced activation in some plant species [34–36]. For example, in the presence of homologous powdery mildew effector Avra10, CNL and MLA10 were transferred to the nucleus and interacted with WRKY and MYB6 transcription factors to further activate the defense response in barley [37]. However, a number of recent studies have demonstrated that coordinated nucleo-cytoplasmic transportation of plant NLRs is required for the full activation of defense response, suggesting that a single NLR protein may activate distinct signaling pathways in the cytoplasm and nucleus [38]. Subcellular localization of peach NLR proteins were predicted using an online tool (https://www.genscript.com/wolf-psort.html). A total of 1289 results were predicted, including 20% in cytoplasm, 17% in plasma membrane, 15% in chloroplast, and relatively few in other organelles (Fig. 4A). In this study, three different types of NLR genes (CNL: *Prupe.2G274900*, TNL: *Prupe.6G152300*, RNL: *Prupe.7G138800*), which had the closest phylogenetic relationship with three types *Arabidopsis thaliana* NLR genes respectively were cloned into the *pCAMBIA1300* vector fused with GFP reporter. The results showed that all three types of NLR were localized both in nucleus and cytoplasm (Fig. 4B), which was consistent with previous study.

### Promoter element analysis of peach NLR genes

**Fig. 5:**
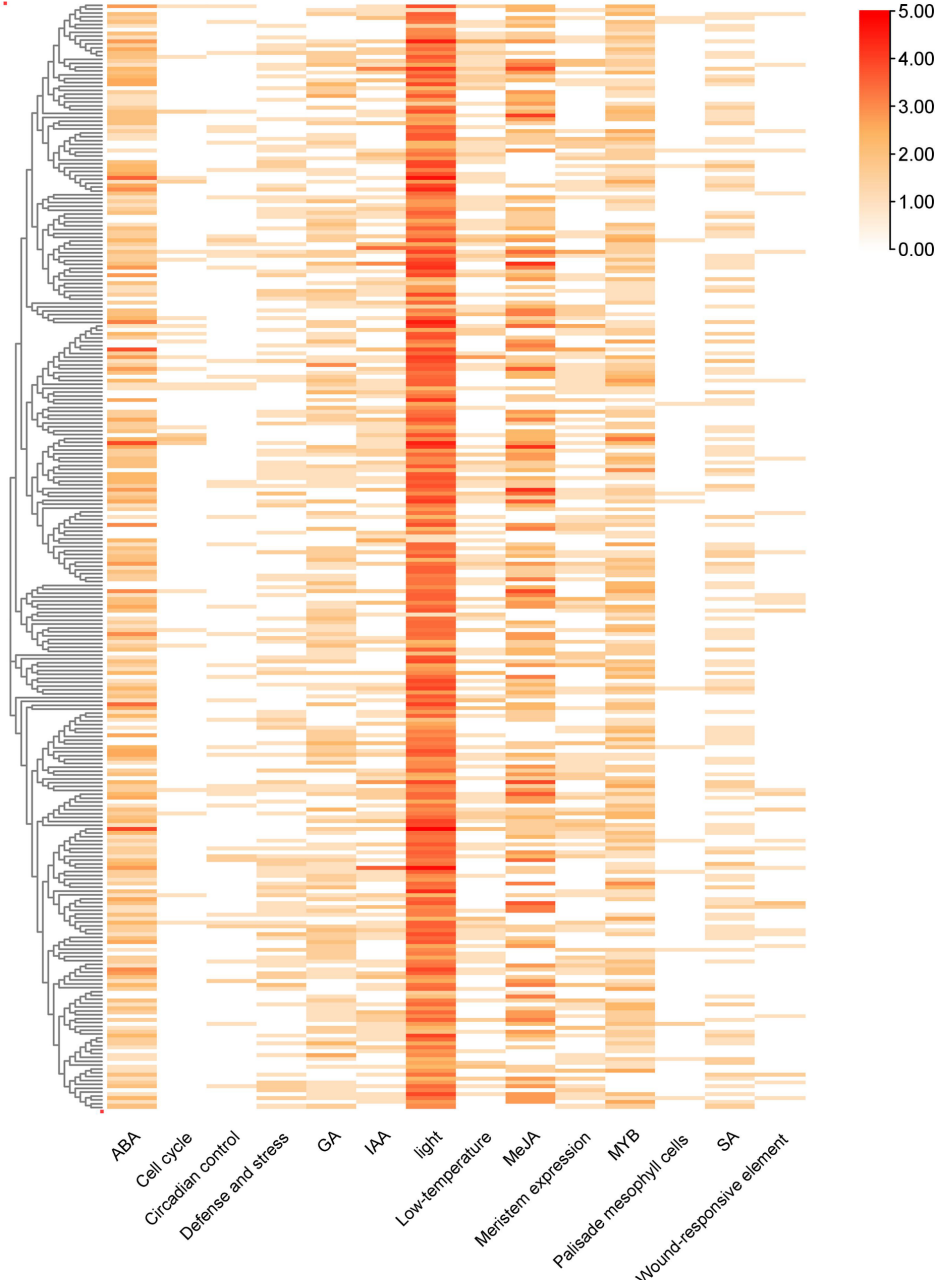
Promoter cis-elements analysis of peach NLR genes. Different color represents the number of cis-elements

The cis-elements in the promoter sequences of 286 NLR genes were predicted using PLANTCARE database (http://bioinformatics.psb.ugent.be/webtools/plantcare/html/). Totally, 14 types of cis-elements were mainly enriched in these promoters, including 5 types of plant hormones response elements (ABA, GA, MeJA, IAA, SA), 3 types of stress response elements (defense and stress, low-temperature, wound-responsive element) and 6 types of growth related response elements (Circadian control, light, Cell cycle, MYB, Meristem expression, Palisade mesophyll cells) (Fig. S4). Among the total elements, plant hormone elements accounted for 35.3%, stress response elements accounted for 48.6%, and growth related elements accounted for 16.1%. In addition, heat map showing the number of cis-elements in each NLR genes was further constructed, and the results showed that the most enriched cis-element was light. Hormone associated element were also greatly enriched in these promoters, such as MeJA, ABA, SA, which indicated that NLR might participate in stress triggered signaling pathways. However, no significant differences in the number and distribution of promoter elements between different subfamilies were found (Fig. 5).

### NLR genes response to aphids and its expression patterns in different tissues

**Fig. 6:**
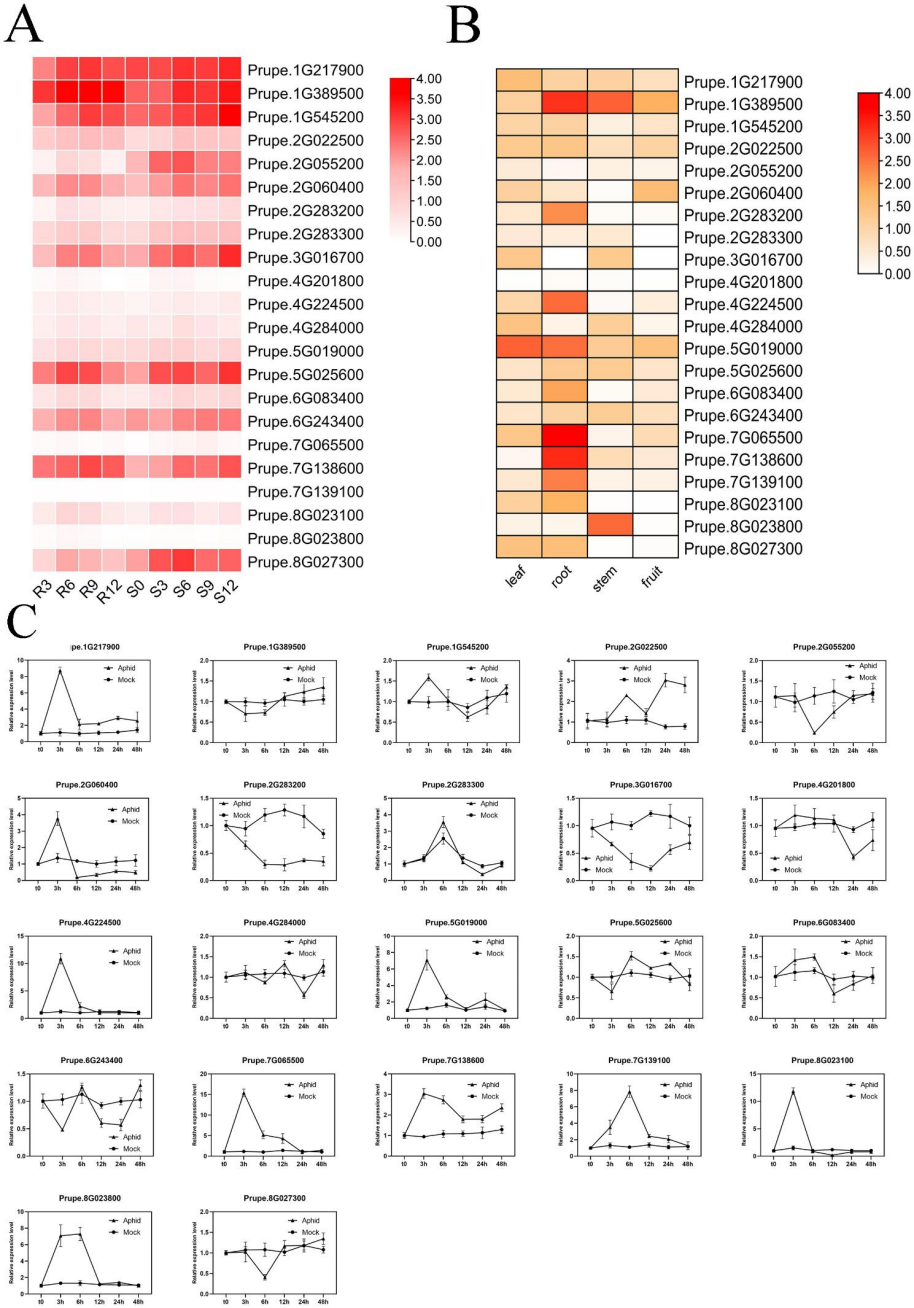
Gene expression analysis of peach NLR. **A** Heat map of differentially expressed peach NLR genes during aphid infestation. Different colors represent the relative expression levels of genes. **B** Relative expression of peach NLR genes in different tissues. **C** Relative expression of peach NLR genes at different stages after aphid infestation

Among the three dominant loci, a strong candidate gene responsible for the dominant GPA resistance in Rm3 locus were identified [29]. However, there is little known about the underlying genes of NLR genes. In this study, to understand the expression patterns of peach NLR genes during aphid infestation, transcriptome analysis was carried out again using the published data [29]. Twenty-two NLRs were significantly up-regulated after aphid infestation. Among them, 8 genes (*Prupe.1G217900, Prupe.1G389500, Prupe.1G545200, Prupe.3G016700, Prupe.5G256000, Prupe.6G243400, Prupe.7G138600, Prupe.8G027300*) showed much higher expression levels than the others (Fig. 6A). Tissue specific analysis of these 22 NLR genes were performed, which showed that they were highly expressed in leaf, stem and root, but little in fruit. This result was consistent with their function in disease and insect resistance (Fig. 6B).

In order to clarify the role of these 22 NLR genes in aphid resistance process, leaf samples at 0 h, 3 h, 6 h, 12 h, 24 h, 48 h after aphid infestation and were collected. As a control, the leaf samples at 0 h, 3 h, 6 h, 12 h, 24 h, 48 h without any processing were also collected. The expression levels were determined by qRT-PCR. The results showed that, most of genes were highly expressed at 3 h (*Prupe.1G217900, Prupe.2G060400, Prupe.2G283300, Prupe.4G224500, Prupe.5G019000, Prupe.7G065500, Prupe.7G138600, Prupe.7G139100, Prupe.8G023100, Prupe.8G023800*), and then rapidly decreased to the normal level. Only a few genes showed lower expression (*Prupe.2G283200, Prupe.3G016700, Prupe.8G027300*). The expression of most genes not infected by aphids did not change significantly with time, only 3 genes (*Prupe.2G283200*, *Prupe.2G283300*, *Prupe.3G016700*) were highly expressed (Fig. 6C). Furthermore, most of the genes showed higher expression levels at the early stage after aphid feeding and then declined to the normal level (Fig. 6C). Such expression pattern might be an appropriate manner for plant immune system to ensure plant self-protection.

## Discussion

Plant immune system play great roles in protecting cells or tissues from pathogen infection through PTI and/or ETI pathways [39, 40]. Insects could produce and release salivary proteins into host cells to further activate ETI system, such as aphid or brown planthopper [21, 41]. Over the past few decades, NLRs were isolated from plants, which could resist various pathogens, including bacteria, fungi, viruses, nematodes and insects [12]. Peach is cultivated worldwide, due to the high nutrition and economic value. However, most of peach cultivars are susceptible to aphids, especially GPA [27]. Besides, the NLR genes in the peach genome have not been systematically analyzed and classified. In this study, a total of 286 NLR genes were identified on peach genome. The bioinformatics analysis and expression of NLR genes during aphid infestation process were analyzed, which provided a foundation for further study on illustrating the mechanism of aphid resistance.

The analysis of the distribution of NLR gene showed that most of them were clustered on the chromosome and clustered at a small area of the chromosome (Fig. 1). For example, dozens of NLR genes are only concentrated in two to three positions on Chr.1, Chr.2, Chr.3, Chr.6, Chr.7 and Chr.8. In previous study, The distribution of NLR genes on chromosomes showed that most of NLR genes exist in clusters and only a few genes exist in single gene loci, which were consistent with the analysis in *Arabidopsis thaliana* [15]. There are two mechanisms for the formation of NLR gene clusters: one is a gene cluster formed by multiple tandem replications of ancestral genes. Such gene clusters composed of closely related genes are considered to be homogeneous clusters. The other is the gene cluster formed by genes with distant genetic relationship or even belonging to different categories (TNL and nTNL, respectively) clustered in adjacent positions due to various mechanisms, such as translocation or ectopic replication [42, 43].

The NLR genes have been reported in many plant species, such as *Vitis vinifera* (535), *Oryza sativa* (508), *Glycine max* (429), *Solanum tuberosum* (438), *Populus* (416), *Gossypium spp* (355) [44, 45]. Phylogenetic analysis showed that the 286 identified NLR genes were divided into four subfamilies according to the differences in N-terminal domain, which were consistent with previous reports (Fig. 2) [15]. According to the results of phylogenetic tree and conservative domain analysis, the NLR genes contained 93 TNLs, 134 CNLs, 11 RNLs and 48 NLs. Among them, NL is divided into NBS_TIR_-LRR, NBS_CC_-LRR and NBS-LRR (Table 1), which is caused by the deletion of CC and TIR domain at the N-terminal [15]. Similar to soybean, the TNL genes in peach are much less than those of CNL, because the evolution rates of TNL and CNL are different [46].

**Table 1:** The number of different types of NLR gens in peach

Predicted Protein	Letter Code	No.
TIR-NBS-LRR	TNL	93
CC-NBS-LRR	CNL	134
RPW8-NBS-LRR	RNL	11
NBS_TIR_-LRR	NL	12
NBS_CC_-LRR	NL	14
NBS-LRR	NL	22
Total		286

The localization of plant NLR proteins might be associated with the localization of effectors [47]. In general, the activation of NLR proteins occurs in the nucleus. For example, in the presence of powdery mildew effector AvrA10, the CNL protein MLA10 in barley needs to be transferred into nucleus to interact with WRKY and MYB6 to further activate the downstream defense signaling [34, 37]. In addition, some plant NLR proteins were not located in nucleus during resistance response process. For example, a CNL protein recognizing potato virus X (PVX) was located in both nucleus and cytoplasm [38, 48]. In present study, subcellular localization of three representative genes in subfamilies I- III showed that CNL, TNL and RNL could localized in both nucleus and cytoplasm (Fig. 4). Pathogen recognition and resistance occurred in cytoplasm [45, 49], indicating their potential function in pathogen resistance.

Most of plant immune responses are accompanied with the release of phytohormones [47]. Salicylic acid (SA) participates in the process of ETI, possibly through the activation of genes involved in cell death [50]. In addition, jasmonic acid (JA) is also involved in plant immune system. There is a complex crosstalk between SA and JA [51]. Analysis of cis-elements in promoters of peach NLR genes identified considerable elements enriched in plant hormone, such as SA, GA, ABA, and MeJA. JA was involved in the regulation of balancing plant growth and disease resistance (Fig. 5) [52]. Therefore, peach NLR genes that response to JA might also have such functions, which provided valuable resources for illustrating the balance of growth and resistance.

The formation of pest resistance is a complex process and a highly coordinated developmental process. Plants have developed a variety of insect resistance mechanisms to decrease pests survival, growth, development, and reproduction [53]. GPA is one of the most dominant aphids, affecting peach growth. During GPA infestation, a large number of NLR genes were activated significantly (Fig. 6C). For example, *Prupe.1G217900*, *Prupe.1G545200*, *Prupe.2G060400*, *Prupe.4G224500*, *Prupe.5G019000*, *Prupe.7G065500*, *Prupe.7G138600*, *Prupe.8G023100* and *Prupe.8G023800* were highly expressed after 3 h of GPA infection. *Prupe.2G022500*, *Prupe.2G283300*, *Prupe.5G025600* and *Prupe.7G13910*0 were highly expressed after 6 or 12 h GPA infection. Tissue specific expression analysis showed that peach NLR genes was mainly expressed in root, leaf and stem, indicating their roles in disease and insect resistance (Fig. 6B). The differentially expressed NLR genes identified during GPA infestation might be useful in illustrating the mechanism of aphid resistance in peach.

## Materials and methods

### Identification of putative peach NLR genes

The protein sequences of NLR genes in *Arabidopsis thaliana* were obtained from NCBI (National Center for Biotechnology Information). Using NLR genes in *Arabidopsis thaliana* as queries, the homologous NLR genes in peach were identified using Blastp tools in NCBI and the NBC and LRR domains were checked manually to get the final set of peach NLR genes. Structural domains were analyzed using Pfam (http://pfam-legacy.xfam.org/) [54]. Physicochemical properties were analyzed and characterized using TBtools software [55].

### Gene distribution

The peach genome annotation file (Ppersica_298_v2.1.gene, https://phytozome-next.jgi.doe.gov/) and chromosome length information were download from JGI (Joint Genomics Institute, http://jgi.doe.gov/). The chromosome annotation of peach NLR gene family members were extracted using TBtools and mapped on chromosome [55].

### Phylogenetic relationships gene structure, motif and collinearity analysis

The phylogenetic tree of peach NLRs was constructed using MEGA11 software, and was viewed using evolview online website (http://www.evolgenius.info/evolview/) [56]. Chromosome information of peach NLR genes were extracted from the peach genome annotation file and were converted into a readable BED file by GSDS2.0 (gene Structure Display Server 2.0 (http://gsds.gao-lab.org/) [57]. Gene structure was further viewed using GSDS2.0. MEME 5.4.1 (https://meme-suite.org/meme/tools/meme) was used to predict and analyze the conserved protein motifs [58]. The base sequence value was set to 15 and other parameters were set with default values. The structure of the conserved protein motifs was plotted using TBtools [55]. Genome-wide collinearity between peach and *Arabidopsis thaliana* were analyzed using MCScanX software and mapped using TBtools [59].

### Subcellular localization analysis

Three peach NLR genes represent the main types of TNL, CNL and RNL were selected according to the phylogenetic tree. Their CDS sequences were obtained from NCBI and were cloned into *pCAMBIA1300* vector fused with GFP under *CaMV 35 S promoter* (*35 S:Prupe.2G274900-GFP*, *35 S:Prupe.6G152300-GFP* and *35 S:Prupe.7G138800-GFP*), Then, the recombined constructs were transferred into Agrobacterium tumefaciens GV3101 for transient overexpression in tobacco leaves using previously described methods [60]. GFP reporter was viewed using a confocal laser microscope (Zeiss LSM880, Germany). Primers used in this section are listed in Table S3.

### Promoter cis-element analysis

The promoter sequences of 286 peach NLR genes (2 kb upstream of the 5’UTR) were download from Genome Database for Rosaceae (https://www.rosaceae.org/) and submitted to PLANTCARE database for promoter element prediction [61]. Their distribution and heat map were plotted using TBtools.

### RNA-seq analysis

The comparative transcriptome data were generated in previous study and the clean reads under SRP144490 were download from NCBI [29]. Clean reads were mapped to reference peach genome (release version 2.0_a2.1) using tophat. The FPKM (fragments per kilobase of exon per million reads mapped) and differentially expressed genes were calculated using cufflink [62].

### RNA extraction and gene expression analysis

Aphid-resistant cultivar, ‘Zao You Tao’, was used for gene expression analysis. To mimic aphid infestation,10 aphids were put on the new young leaves and bagged with 100-mesh insect screens to avoid aphid escaping. Leaf samples were collected at 0 h, 3 h, 6 h, 12 h, 24 h, 48 h after infestation and immediately frozen in liquid nitrogen and then stored at -80℃ for analysis. Total RNA was extracted from the samples using an RNA extraction kit (Tiangen, China) and first-strand cDNA was synthesized using PrimeScript first-strand cDNA synthesis kit (Takara, Dalian, China). Real-time quantitative polymerase chain reaction (qRT-PCR) was performed on ABI7500 system using SYBR premix ExTaq (Takara, China) with the following procedure: 95 °C for 5 min, followed by 45 cycles at 95 °C for 10 s, 58 °C for 10 s and 72 °C for 20 s. The relative expression level was calculated by 2^−ΔΔCT^ method [63]. Primers for qRT-PCR are listed in Table S3.

### Electronic supplementary material

Below is the link to the electronic supplementary material.

Supplementary Material 1

## Data Availability

All the data generated or analyzed during this study are included in this published article and its supplementary information fles. The peach sequences in this article can be found from phytozome (Phytozome (doe. ov)). The *Arabidopsis thaliana* sequences and the transcriptome data in this article were downloaded from NCBI (National Center for Biotechnology Information). All plant materials were selected from peach provided by the F. Peng’s lab, Shandong Agricultural University, Taian, China.

## References

[1] Boller T, Felix G (2009). A renaissance of elicitors: perception of microbe-associated molecular patterns and danger signals by pattern-recognition receptors. Annu Rev Plant Biol.

[2] Maekawa T, Kufer TA, Schulze-Lefert P (2011). NLR functions in plant and animal immune systems: so far and yet so close. Nat Immunol.

[3] Chisholm ST, Coaker G, Day B, Staskawicz BJ (2006). Host-microbe interactions: shaping the evolution of the plant immune response. Cell.

[4] Feng F, Zhou J-M (2012). Plant–bacterial pathogen interactions mediated by type III effectors. Curr Opin Plant Biol.

[5] Meyers BC, Dickerman AW, Michelmore RW, Sivaramakrishnan S, Sobral BW, Young ND (1999). Plant disease resistance genes encode members of an ancient and diverse protein family within the nucleotide-binding superfamily. Plant J.

[6] Collier SM, Hamel L-P, Moffett P (2011). Cell death mediated by the N-terminal domains of a unique and highly conserved class of NB-LRR protein. Mol Plant Microbe Interact.

[7] Xiao S, Ellwood S, Calis O, Patrick E, Li T, Coleman M, Turner JG (2001). Broad-spectrum mildew resistance in Arabidopsis thaliana mediated by RPW8. Science.

[8] Bonardi V, Tang S, Stallmann A, Roberts M, Cherkis K, Dangl JL (2011). Expanded functions for a family of plant intracellular immune receptors beyond specific recognition of pathogen effectors. Proc Natl Acad Sci.

[9] Xue J-Y, Wang Y, Wu P, Wang Q, Yang L-T, Pan X-H, Wang B, Chen J-Q (2012). A primary survey on bryophyte species reveals two novel classes of nucleotide-binding site (NBS) genes. PLoS ONE.

[10] Castel B, Ngou PM, Cevik V, Redkar A, Kim DS, Yang Y, Ding P, Jones JD (2019). Diverse NLR immune receptors activate defence via the RPW 8-NLR NRG 1. New Phytol.

[11] Liu Y, Zhang Y-M, Tang Y, Chen J-Q, Shao Z-Q (2023). The evolution of plant NLR immune receptors and downstream signal components. Curr Opin Plant Biol.

[12] Gao Y, Wang W, Zhang T, Gong Z, Zhao H, Han G-Z (2018). Out of water: the origin and early diversification of plant R-genes. Plant Physiol.

[13] McHale L, Tan X, Koehl P, Michelmore RW (2006). Plant NBS-LRR proteins: adaptable guards. Genome Biol.

[14] Fenyk S, Campillo ASE, Pohl E, Hussey PJ, Cann MJ (2012). A nucleotide phosphatase activity in the nucleotide binding domain of an orphan resistance protein from rice. J Biol Chem.

[15] Meyers BC, Kozik A, Griego A, Kuang H, Michelmore RW (2003). Genome-wide analysis of NBS-LRR–encoding genes in Arabidopsis. Plant Cell.

[16] Van Ooijen G, Mayr G, Kasiem MM, Albrecht M, Cornelissen BJ, Takken FL (2008). Structure–function analysis of the NB-ARC domain of plant disease resistance proteins. J Exp Bot.

[17] Steele JF, Hughes RK, Banfield MJ (2019). Structural and biochemical studies of an NB-ARC domain from a plant NLR immune receptor. PLoS ONE.

[18] Russell AR, Ashfield T, Innes RW (2015). Pseudomonas syringae effector AvrPphB suppresses AvrB-induced activation of RPM1 but not AvrRpm1-induced activation. Mol Plant Microbe Interact.

[19] Zhang H, Li S, Wang F, Xiang J, Li F (2020). Identification and functional study of an LRR domain containing membrane protein in Litopenaeus vannamei. Dev Comp Immunol.

[20] Bober M, Mörgelin M, Olin AI, von Pawel-Rammingen U, Collin M (2011). The membrane bound LRR lipoprotein Slr, and the cell wall-anchored M1 protein from Streptococcus pyogenes both interact with type I collagen. PLoS ONE.

[21] Dangl JL, Horvath DM, Staskawicz BJ (2013). Pivoting the plant immune system from dissection to deployment. Science.

[22] Haverkort AJ, Struik P, Visser R, Jacobsen E (2009). Applied biotechnology to combat late blight in potato caused by Phytophthora infestans. Potato Res.

[23] Nombela G, Williamson VM, Muñiz M (2003). The root-knot nematode resistance gene Mi-1.2 of tomato is responsible for resistance against the whitefly Bemisia tabaci. Mol Plant Microbe Interact.

[24] Nicolis V, Venter E (2018). Silencing of a unique integrated domain nucleotide-binding leucine-rich repeat gene in wheat abolishes Diuraphis noxia resistance. Mol Plant Microbe Interact.

[25] Wang F, Zhao S, Han Y, Shao Y, Dong Z, Gao Y, Zhang K, Liu X, Li D, Chang J (2013). Efficient and fine mapping of RMES1 conferring resistance to sorghum aphid Melanaphis sacchari. Mol Breeding.

[26] Cevik V, King GJ (2002). Resolving the aphid resistance locus Sd-1 on a BAC contig within a sub-telomeric region of Malus linkage group 7. Genome.

[27] Wang Z, Wu X, Zhang B, Xiao Y, Guo J, Liu J, Chen Q, Peng F (2023). Genome-wide identification, bioinformatics and expression analysis of HD-Zip gene family in peach. BMC Plant Biol.

[28] Bos JI, Prince D, Pitino M, Maffei ME, Win J, Hogenhout SA (2010). A functional genomics approach identifies candidate effectors from the aphid species Myzus persicae (green peach aphid). PLoS Genet.

[29] Pan L, Lu Z, Yan L, Zeng W, Shen Z, Yu M, Bu L, Cui G, Niu L, Wang Z (2022). NLR1 is a strong candidate for the Rm3 dominant green peach aphid (Myzus persicae) resistance trait in peach. J Exp Bot.

[30] Sarris PF, Cevik V, Dagdas G, Jones JD, Krasileva KV (2016). Comparative analysis of plant immune receptor architectures uncovers host proteins likely targeted by pathogens. BMC Biol.

[31] Kroj T, Chanclud E, Michel-Romiti C, Grand X, Morel JB (2016). Integration of decoy domains derived from protein targets of pathogen effectors into plant immune receptors is widespread. New Phytol.

[32] Guo Y-L, Fitz J, Schneeberger K, Ossowski S, Cao J, Weigel D (2011). Genome-wide comparison of nucleotide-binding site-leucine-rich repeat-encoding genes in Arabidopsis. Plant Physiol.

[33] Ossowski S, Schneeberger K, Clark RM, Lanz C, Warthmann N, Weigel D (2008). Sequencing of natural strains of Arabidopsis thaliana with short reads. Genome Res.

[34] Shen Q-H, Saijo Y, Mauch S, Biskup C, Bieri S, Keller B, Seki H, Ülker B, Somssich IE, Schulze-Lefert P. Nuclear activity of MLA immune receptors links isolate-specific and basal disease-resistance responses. *science* 2007, 315(5815):1098–1103. 10.1126/science.1136372 17185563

[35] Wirthmueller L, Zhang Y, Jones JD, Parker JE (2007). Nuclear accumulation of the Arabidopsis immune receptor RPS4 is necessary for triggering EDS1-dependent defense. Curr Biol.

[36] Caplan JL, Mamillapalli P, Burch-Smith TM, Czymmek K (2008). Dinesh-Kumar Sá: chloroplastic protein NRIP1 mediates innate immune receptor recognition of a viral effector. Cell.

[37] Chang C, Yu D, Jiao J, Jing S, Schulze-Lefert P, Shen Q-H (2013). Barley MLA immune receptors directly interfere with antagonistically acting transcription factors to initiate disease resistance signaling. Plant Cell.

[38] Tameling WI, Baulcombe DC (2007). Physical association of the NB-LRR resistance protein rx with a ran GTPase–activating protein is required for extreme resistance to Potato virus X. Plant Cell.

[39] Porter BW, Paidi M, Ming R, Alam M, Nishijima WT, Zhu YJ (2009). Genome-wide analysis of Carica papaya reveals a small NBS resistance gene family. Mol Genet Genomics.

[40] Ngou BPM, Ahn H-K, Ding P, Jones JD (2021). Mutual potentiation of plant immunity by cell-surface and intracellular receptors. Nature.

[41] Bent AF, Mackey D (2007). Elicitors, effectors, and R genes: the new paradigm and a lifetime supply of questions. Annu Rev Phytopathol.

[42] Leister D (2004). Tandem and segmental gene duplication and recombination in the evolution of plant disease resistance genes. Trends Genet.

[43] Zhang YM, Shao ZQ, Wang Q, Hang YY, Xue JY, Wang B, Chen JQ (2016). Uncovering the dynamic evolution of nucleotide-binding site‐leucine‐rich repeat (NBS‐LRR) genes in Brassicaceae. J Integr Plant Biol.

[44] Li J, Ding J, Zhang W, Zhang Y, Tang P, Chen J-Q, Tian D, Yang S (2010). Unique evolutionary pattern of numbers of gramineous NBS–LRR genes. Mol Genet Genomics.

[45] Qi D, Innes RW (2013). Recent advances in plant NLR structure, function, localization, and signaling. Front Immunol.

[46] Zhang X, Feng Y, Cheng H, Tian D, Yang S, Chen J-Q (2011). Relative evolutionary rates of NBS-encoding genes revealed by soybean segmental duplication. Mol Genet Genomics.

[47] Liang X, Zhou J-M (2018). Receptor-like cytoplasmic kinases: central players in plant receptor kinase–mediated signaling. Annu Rev Plant Biol.

[48] Sacco MA, Mansoor S, Moffett P (2007). A RanGAP protein physically interacts with the NB-LRR protein rx, and is required for Rx‐mediated viral resistance. Plant J.

[49] Heidrich K, Wirthmueller L, Tasset C, Pouzet C, Deslandes L, Parker JE (2011). Arabidopsis EDS1 connects pathogen effector recognition to cell compartment–specific immune responses. Science.

[50] Radojičić A, Li X, Zhang Y (2018). Salicylic acid: a double-edged sword for programed cell death in plants. Front Plant Sci.

[51] Glazebrook J (2005). Contrasting mechanisms of defense against biotrophic and necrotrophic pathogens. Annu Rev Phytopathol.

[52] Guo J, Wang H, Guan W, Guo Q, Wang J, Yang J, Peng Y, Shan J, Gao M, Shi S (2023). A tripartite rheostat controls self-regulated host plant resistance to insects. Nature.

[53] Smith CM. Plant resistance to arthropods: molecular and conventional approaches. Springer; 2005.

[54] Bateman A, Coin L, Durbin R, Finn RD, Hollich V, Griffiths-Jones S, Khanna A, Marshall M, Moxon S, Sonnhammer EL (2004). The pfam protein families database. Nucleic Acids Res.

[55] Chen C, Chen H, Zhang Y, Thomas HR, Frank MH, He Y, Xia R (2020). TBtools: an integrative toolkit developed for interactive analyses of big biological data. Mol Plant.

[56] Tamura K, Stecher G, Kumar S (2021). MEGA11: molecular evolutionary genetics analysis version 11. Mol Biol Evol.

[57] Hu B, Jin J, Guo A-Y, Zhang H, Luo J, Gao G (2015). GSDS 2.0: an upgraded gene feature visualization server. Bioinformatics.

[58] Bailey TL, Boden M, Buske FA, Frith M, Grant CE, Clementi L, Ren J, Li WW, Noble WS (2009). MEME SUITE: tools for motif discovery and searching. Nucleic Acids Res.

[59] Wang Y, Tang H, DeBarry JD, Tan X, Li J, Wang X, Lee T-h, Jin H, Marler B, Guo H (2012). MCScanX: a toolkit for detection and evolutionary analysis of gene synteny and collinearity. Nucleic Acids Res.

[60] Sparkes IA, Runions J, Kearns A, Hawes C (2006). Rapid, transient expression of fluorescent fusion proteins in tobacco plants and generation of stably transformed plants. Nat Protoc.

[61] Lescot M, Déhais P, Thijs G, Marchal K, Moreau Y, Van de Peer Y, Rouzé P, Rombauts S (2002). PlantCARE, a database of plant cis-acting regulatory elements and a portal to tools for in silico analysis of promoter sequences. Nucleic Acids Res.

[62] Trapnell C, Pachter L, Salzberg SL (2009). TopHat: discovering splice junctions with RNA-Seq. Bioinformatics.

[63] Livak KJ, Schmittgen TD (2001). Analysis of relative gene expression data using real-time quantitative PCR and the 2 – ∆∆CT method. Methods.

