# Supplementary material for: Genome-wide identification of nucleotide-binding domain leucine-rich repeat (NLR) genes and their association with green peach aphid (Myzus persicae) resistance in peach
Source: BMC Plant Biol. 2023 Oct 25;23:513. doi: 10.1186/s12870-023-04474-7 (PMC10598982; doi:10.1186/s12870-023-04474-7)
Supplement: Supplementary file 1 — Supplementary Material 1 [file 12870_2023_4474_MOESM1_ESM.docx]

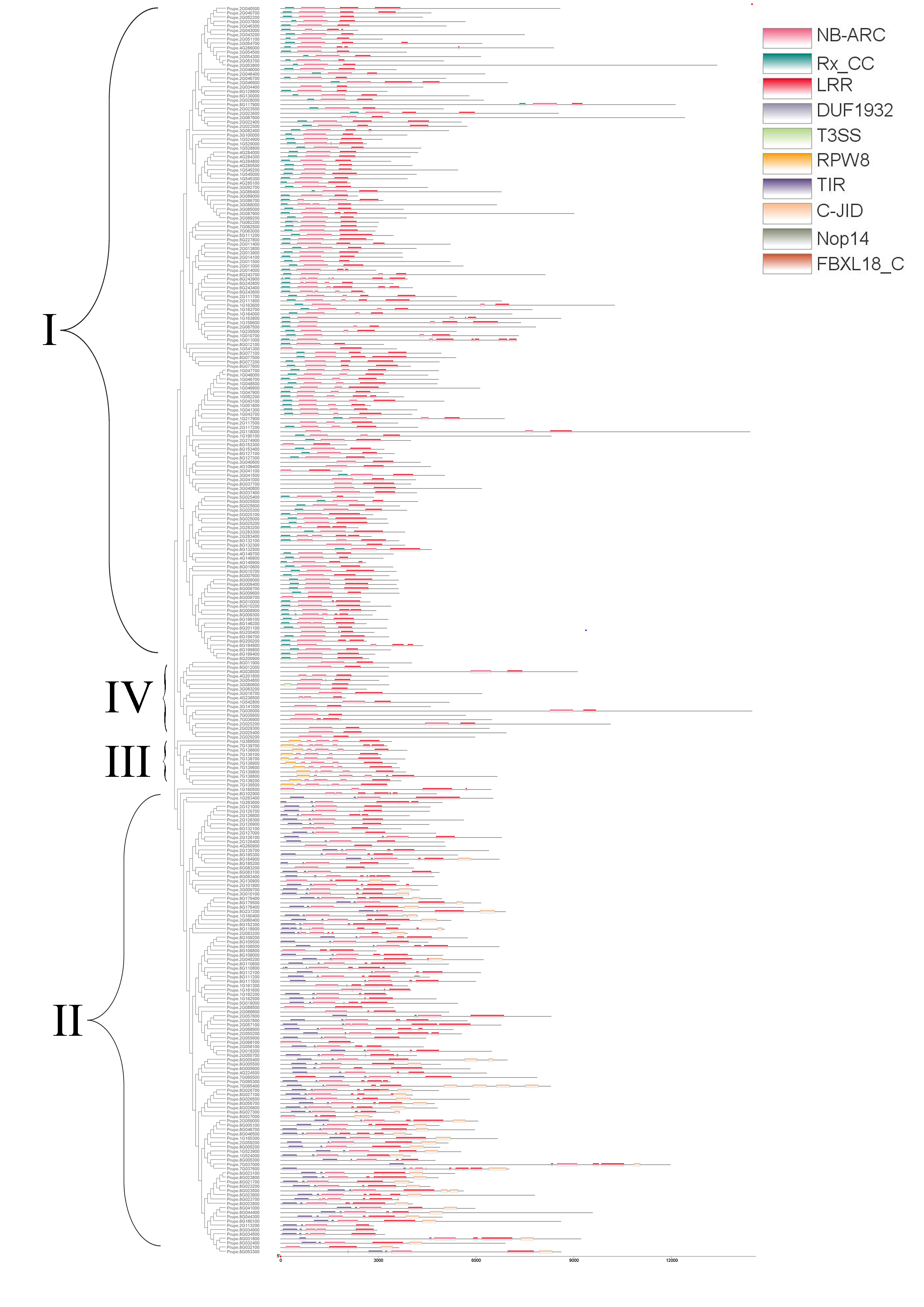


Fig. S1 Conserved domain analysis of subfamilies. Boxes of different colors represent different domains of peach NLR proteins. The horizontal line represents the amino acid sequence. The I, II, III and IV represents subfamily I, II,III and IV of peach NLR genes.


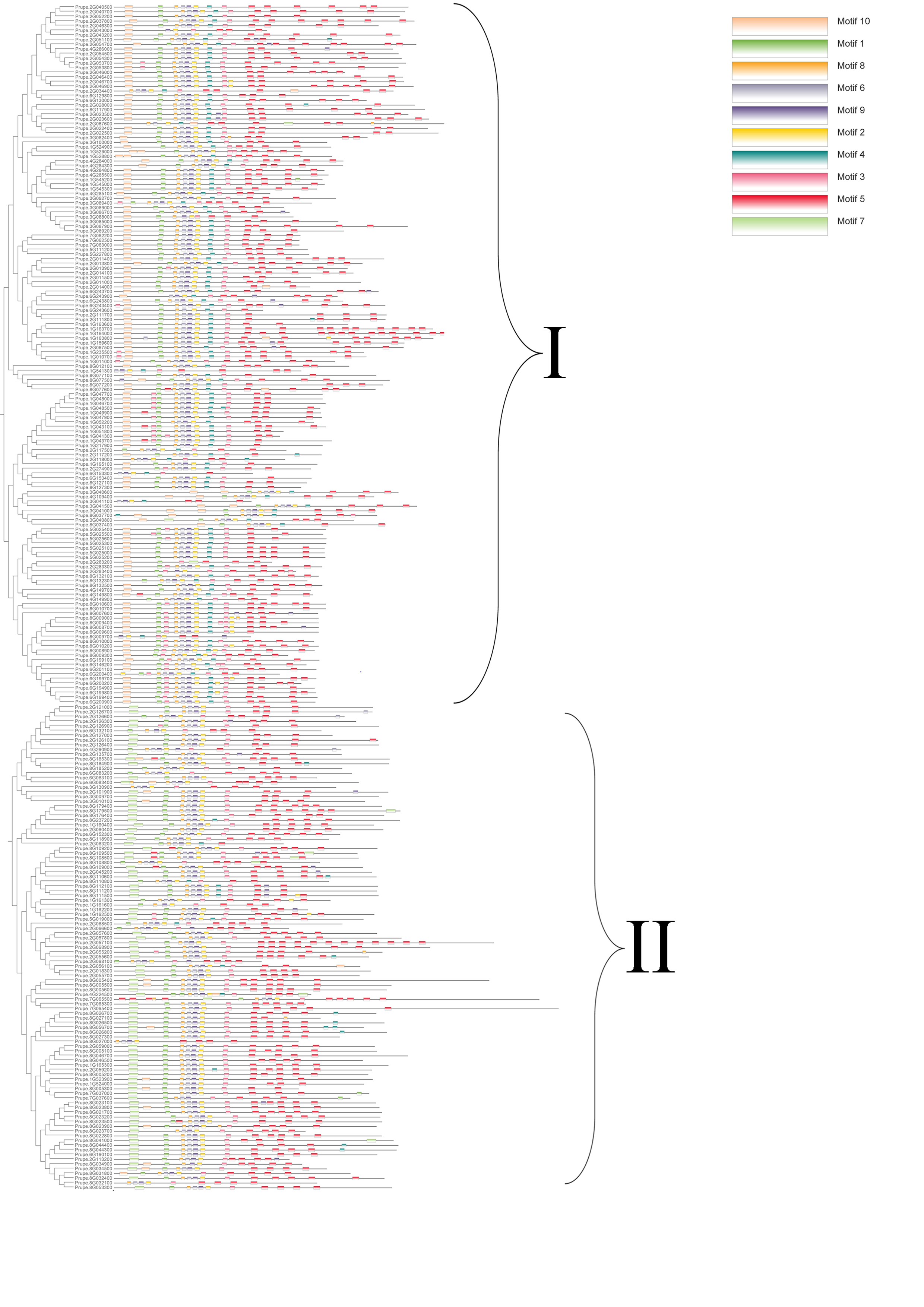


Fig. S2 Motif analysis of subfamily I, II. Boxes of different colors represent different motifs. The horizontal line represents the amino acid sequence. The I and II represents subfamily I and IV of peach NLR genes.


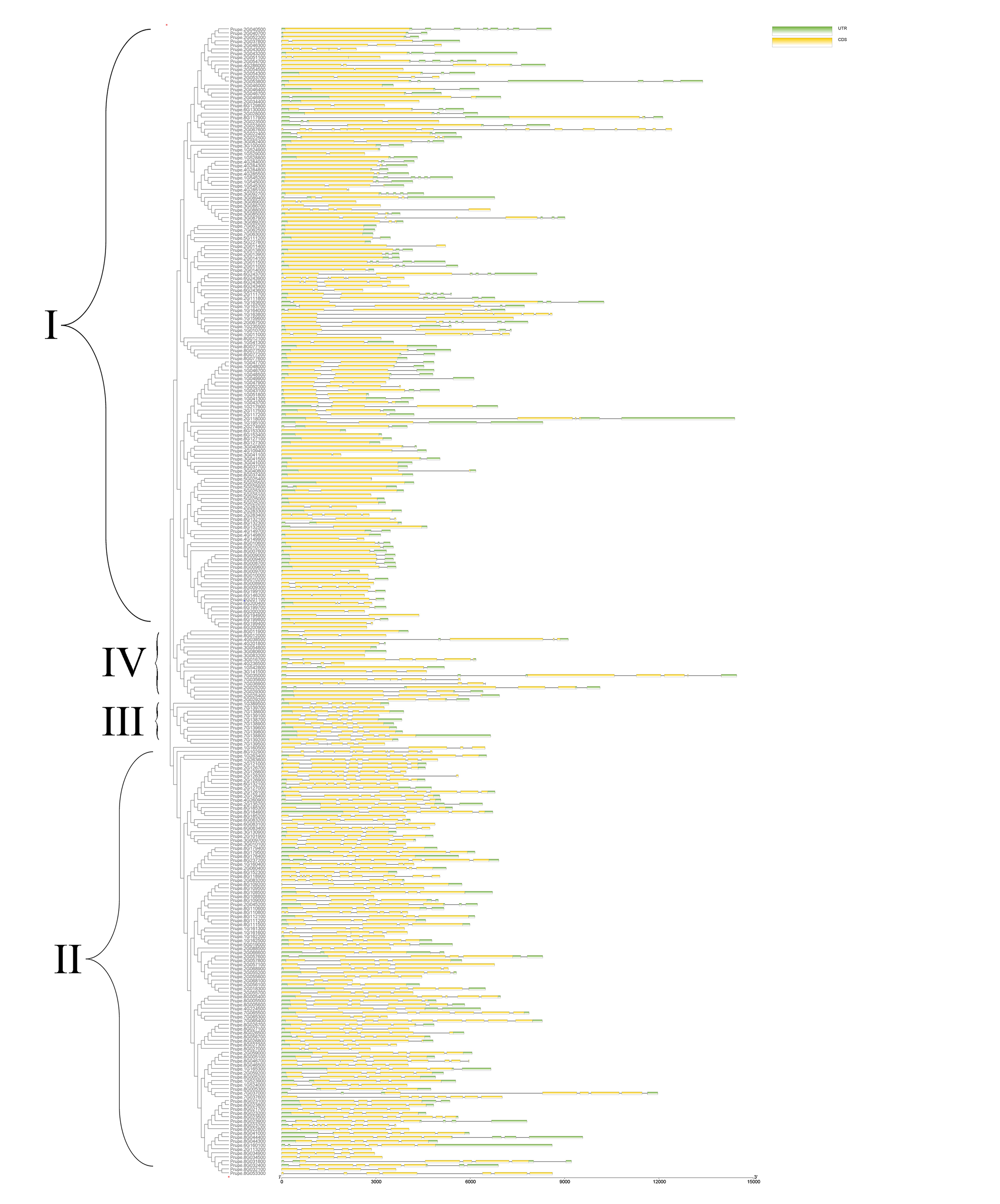


Fig. S3 Gene structure analysis of subfamilies. Boxes of different colors represent different structure of peach NLR genes. The horizontal line represents the base sequence. The I, II, III and IV represents subfamily I, II,III and IV of peach NLR genes.


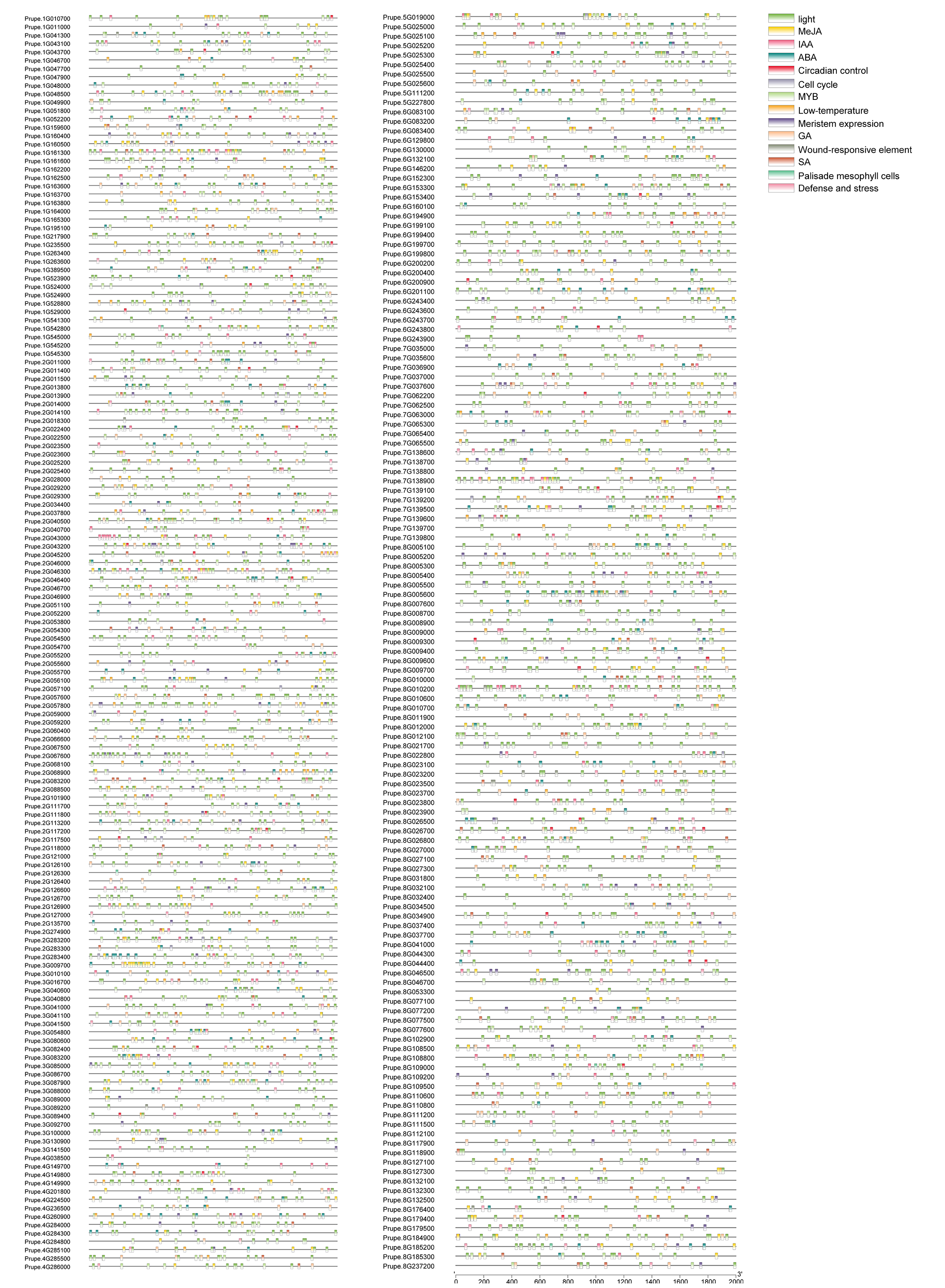


Fig. S4 Cis-elements in the promoter of peach NLR genes. Boxes of different colors represent different cis-elements. The horizontal line represents the promoter base sequence of peach NLR genes.

**Table S1** Physical and chemical properties of NLR gene in peach.

| Gene ID | AA | MW(Da) | PI | Instability Index | Aliphatic Index | GAH | Chrom |
| --- | --- | --- | --- | --- | --- | --- | --- |
| Prupe.1G010700 | 1132 | 128506.87 | 6.46 | 47.29 | 101.02 | -0.199 | Chr.1 |
| Prupe.1G011000 | 990 | 111697.82 | 5.58 | 47.21 | 102.7 | -0.202 | Chr.1 |
| Prupe.1G041300 | 743 | 85131.45 | 8.57 | 48.05 | 103.98 | -0.192 | Chr.1 |
| Prupe.1G043100 | 949 | 108679.48 | 6.41 | 45.41 | 102.46 | -0.175 | Chr.1 |
| Prupe.1G043700 | 976 | 111354.26 | 8.69 | 47.68 | 102.06 | -0.123 | Chr.1 |
| Prupe.1G046700 | 948 | 108862.06 | 8.52 | 45.09 | 102.48 | -0.257 | Chr.1 |
| Prupe.1G047700 | 935 | 107500.57 | 8.61 | 44.68 | 100.46 | -0.273 | Chr.1 |
| Prupe.1G047900 | 931 | 106838.03 | 8.62 | 43.32 | 104.22 | -0.22 | Chr.1 |
| Prupe.1G048000 | 935 | 107287.62 | 8.63 | 43.74 | 103.88 | -0.221 | Chr.1 |
| Prupe.1G048500 | 924 | 105619.25 | 8.61 | 45.94 | 103.33 | -0.213 | Chr.1 |
| Prupe.1G049900 | 928 | 106851.02 | 9.19 | 49.09 | 102.68 | -0.276 | Chr.1 |
| Prupe.1G051800 | 759 | 87397.93 | 8.51 | 47.11 | 103.06 | -0.247 | Chr.1 |
| Prupe.1G052200 | 896 | 103411.69 | 7.55 | 48.88 | 102.02 | -0.262 | Chr.1 |
| Prupe.1G159600 | 1302 | 147458.85 | 5.82 | 50.56 | 102.9 | -0.155 | Chr.1 |
| Prupe.1G160400 | 1154 | 130572.34 | 6.18 | 47.27 | 100.42 | -0.116 | Chr.1 |
| Prupe.1G160500 | 853 | 95701.49 | 5.67 | 44 | 101.82 | -0.039 | Chr.1 |
| Prupe.1G161300 | 960 | 108531.41 | 7.47 | 44.22 | 107.29 | 0.04 | Chr.1 |
| Prupe.1G161600 | 711 | 80710.09 | 9 | 49.96 | 104.73 | -0.166 | Chr.1 |
| Prupe.1G162200 | 860 | 96726.47 | 6.08 | 37.37 | 99.4 | -0.143 | Chr.1 |
| Prupe.1G162500 | 1155 | 130468.49 | 6.36 | 42.73 | 99.14 | -0.13 | Chr.1 |
| Prupe.1G163600 | 1049 | 117334.87 | 5.66 | 49.82 | 104.25 | -0.127 | Chr.1 |
| Prupe.1G163700 | 1431 | 161693.39 | 7.81 | 43.16 | 106.38 | -0.08 | Chr.1 |
| Prupe.1G163800 | 1432 | 161625.7 | 6.9 | 44.77 | 104.83 | -0.103 | Chr.1 |
| Prupe.1G164000 | 1556 | 175282.9 | 6.29 | 42.74 | 109.22 | -0.046 | Chr.1 |
| Prupe.1G165300 | 1218 | 138434.66 | 6.46 | 43.33 | 94.41 | -0.199 | Chr.1 |
| Prupe.1G195100 | 911 | 105542.59 | 7.03 | 52.45 | 104.5 | -0.133 | Chr.1 |
| Prupe.1G217900 | 935 | 108150.62 | 8.34 | 46.6 | 102.65 | -0.162 | Chr.1 |
| Prupe.1G235500 | 1120 | 127553.74 | 7.69 | 45.69 | 100.7 | -0.243 | Chr.1 |
| Prupe.1G263400 | 1372 | 153815.65 | 6.79 | 39.39 | 107.3 | -0.102 | Chr.1 |
| Prupe.1G263600 | 1190 | 133502.02 | 6.58 | 41.48 | 108.27 | -0.071 | Chr.1 |
| Prupe.1G389500 | 822 | 92947.37 | 6.18 | 43.37 | 106.05 | -0.052 | Chr.1 |
| Prupe.1G523900 | 1148 | 130322.15 | 7.03 | 42.21 | 95.57 | -0.2 | Chr.1 |
| Prupe.1G524000 | 1109 | 126439.17 | 6.05 | 42.75 | 95.24 | -0.225 | Chr.1 |
| Prupe.1G524900 | 973 | 112433.72 | 5.81 | 47.61 | 98.32 | -0.306 | Chr.1 |
| Prupe.1G528800 | 977 | 112350.42 | 5.46 | 45.42 | 103.61 | -0.273 | Chr.1 |
| Prupe.1G529000 | 844 | 97119.19 | 6.1 | 46.81 | 101.59 | -0.201 | Chr.1 |
| Prupe.1G541300 | 839 | 95110.96 | 6.63 | 51.78 | 99.11 | -0.201 | Chr.1 |
| Prupe.1G542800 | 1041 | 117358.35 | 5.64 | 45.29 | 97.88 | -0.146 | Chr.1 |
| Prupe.1G545000 | 944 | 108464.67 | 5.27 | 39.87 | 97.35 | -0.29 | Chr.1 |
| Prupe.1G545200 | 934 | 107748.2 | 5.79 | 41.89 | 97.25 | -0.298 | Chr.1 |
| Prupe.1G545300 | 909 | 105194.68 | 6.19 | 40.55 | 96.17 | -0.326 | Chr.1 |
| Prupe.2G011000 | 1106 | 126448.5 | 6.45 | 46.2 | 100.24 | -0.217 | Chr.2 |
| Prupe.2G011400 | 1211 | 138623.04 | 7.62 | 42.43 | 102.77 | -0.145 | Chr.2 |
| Prupe.2G011500 | 882 | 101712.12 | 8.44 | 41.12 | 101.45 | -0.268 | Chr.2 |
| Prupe.2G013800 | 1114 | 127687.03 | 6.58 | 43.56 | 103.33 | -0.179 | Chr.2 |
| Prupe.2G013900 | 1047 | 120240.98 | 6.2 | 45.04 | 99.99 | -0.221 | Chr.2 |
| Prupe.2G014000 | 878 | 101404.18 | 7.81 | 49.37 | 99.07 | -0.269 | Chr.2 |
| Prupe.2G014100 | 1073 | 122771.1 | 7.27 | 43.7 | 98.74 | -0.237 | Chr.2 |
| Prupe.2G018300 | 1139 | 130210.83 | 5.74 | 48.03 | 98.98 | -0.214 | Chr.2 |
| Prupe.2G022400 | 1407 | 159345.16 | 6.97 | 44.35 | 100.65 | -0.172 | Chr.2 |
| Prupe.2G022500 | 1455 | 165687.48 | 6.7 | 45.31 | 99.59 | -0.155 | Chr.2 |
| Prupe.2G023500 | 1320 | 148932.78 | 6.3 | 43.49 | 103.31 | -0.191 | Chr.2 |
| Prupe.2G023600 | 1413 | 158967.68 | 6.39 | 40.73 | 103.33 | -0.155 | Chr.2 |
| Prupe.2G025200 | 1410 | 159578.52 | 6.5 | 48.68 | 95.82 | -0.244 | Chr.2 |
| Prupe.2G025400 | 1459 | 165120.89 | 6.17 | 46.67 | 95.65 | -0.251 | Chr.2 |
| Prupe.2G028000 | 1349 | 152907.74 | 6.42 | 48.69 | 105.64 | -0.087 | Chr.2 |
| Prupe.2G029200 | 1415 | 160613.78 | 6.05 | 46.69 | 96.83 | -0.238 | Chr.2 |
| Prupe.2G029300 | 1428 | 161399.67 | 5.93 | 43.28 | 97.24 | -0.223 | Chr.2 |
| Prupe.2G034400 | 1253 | 143160.65 | 5.68 | 54.31 | 97.37 | -0.167 | Chr.2 |
| Prupe.2G037800 | 1347 | 152234.53 | 5.95 | 44.73 | 100.08 | -0.177 | Chr.2 |
| Prupe.2G040500 | 1320 | 148949.78 | 7.87 | 42.28 | 101.61 | -0.171 | Chr.2 |
| Prupe.2G040700 | 1305 | 146739.9 | 6.15 | 41.91 | 103.45 | -0.136 | Chr.2 |
| Prupe.2G043000 | 684 | 78117.73 | 6.1 | 38.13 | 101.89 | -0.177 | Chr.2 |
| Prupe.2G043200 | 1284 | 145430.4 | 6.29 | 42.89 | 101.34 | -0.177 | Chr.2 |
| Prupe.2G045200 | 1145 | 130898.34 | 6.48 | 43.88 | 97.42 | -0.123 | Chr.2 |
| Prupe.2G046000 | 1034 | 117306.32 | 6.21 | 41.75 | 99.54 | -0.215 | Chr.2 |
| Prupe.2G046300 | 1186 | 134835.86 | 6.71 | 43.98 | 97.16 | -0.246 | Chr.2 |
| Prupe.2G046400 | 1296 | 147475.6 | 7.26 | 41.1 | 101.09 | -0.187 | Chr.2 |
| Prupe.2G046700 | 1301 | 147428.95 | 5.83 | 44.16 | 100.48 | -0.162 | Chr.2 |
| Prupe.2G046900 | 1343 | 151845.01 | 6 | 46.21 | 100.24 | -0.164 | Chr.2 |
| Prupe.2G051100 | 1022 | 116705.15 | 8.09 | 40.35 | 98.07 | -0.235 | Chr.2 |
| Prupe.2G052200 | 1281 | 144786.36 | 6.02 | 39.41 | 100.36 | -0.174 | Chr.2 |
| Prupe.2G053700 | 1309 | 148288.8 | 5.98 | 49.51 | 97.2 | -0.236 | Chr.2 |
| Prupe.2G053800 | 1275 | 144086.93 | 6.75 | 43.4 | 101.37 | -0.17 | Chr.2 |
| Prupe.2G054300 | 1290 | 145776.76 | 6.52 | 42.16 | 102.79 | -0.135 | Chr.2 |
| Prupe.2G054500 | 1276 | 144491.39 | 6.89 | 44.9 | 102.28 | -0.149 | Chr.2 |
| Prupe.2G054700 | 1355 | 153418.54 | 6.23 | 46.44 | 102.01 | -0.131 | Chr.2 |
| Prupe.2G055200 | 1191 | 134441.27 | 5.44 | 42.63 | 96.88 | -0.121 | Chr.2 |
| Prupe.2G055600 | 1131 | 127595.9 | 6.11 | 48.12 | 97.11 | -0.115 | Chr.2 |
| Prupe.2G055700 | 1090 | 124509.84 | 6.54 | 49.07 | 98.5 | -0.183 | Chr.2 |
| Prupe.2G056100 | 1092 | 123969.78 | 5.41 | 42.81 | 95.55 | -0.273 | Chr.2 |
| Prupe.2G057100 | 1687 | 190520.77 | 5.62 | 43.43 | 104.15 | -0.076 | Chr.2 |
| Prupe.2G057600 | 1167 | 132265.92 | 7.26 | 40.21 | 101.11 | -0.132 | Chr.2 |
| Prupe.2G057800 | 1276 | 144871.63 | 5.2 | 45.59 | 97.13 | -0.192 | Chr.2 |
| Prupe.2G059000 | 1157 | 131509.66 | 7.43 | 44.76 | 94.32 | -0.23 | Chr.2 |
| Prupe.2G059200 | 1145 | 129864.06 | 6.79 | 47.69 | 100.43 | -0.141 | Chr.2 |
| Prupe.2G060400 | 1195 | 134945.97 | 7.58 | 49.23 | 99.37 | -0.107 | Chr.2 |
| Prupe.2G066600 | 776 | 87916.1 | 6.08 | 45.84 | 107.53 | 0.018 | Chr.2 |
| Prupe.2G067500 | 1298 | 147470.16 | 5.97 | 47.11 | 100.55 | -0.211 | Chr.2 |
| Prupe.2G067600 | 1492 | 169785.86 | 8.63 | 51.21 | 102.27 | -0.183 | Chr.2 |
| Prupe.2G068100 | 654 | 74382.61 | 6.12 | 42.4 | 116.22 | -0.005 | Chr.2 |
| Prupe.2G068900 | 1403 | 158281.39 | 5.84 | 39.69 | 100.3 | -0.155 | Chr.2 |
| Prupe.2G083200 | 752 | 85595.83 | 5.84 | 41.18 | 90.64 | -0.27 | Chr.2 |
| Prupe.2G088500 | 1013 | 114151.92 | 5.86 | 38.55 | 94.46 | -0.199 | Chr.2 |
| Prupe.2G101900 | 1217 | 138908.84 | 6.7 | 42.38 | 89.41 | -0.351 | Chr.2 |
| Prupe.2G111700 | 1222 | 138421.01 | 6.92 | 44.04 | 103.94 | -0.114 | Chr.2 |
| Prupe.2G111800 | 1217 | 137760.93 | 7.25 | 45.05 | 102.6 | -0.15 | Chr.2 |
| Prupe.2G113200 | 778 | 88970.67 | 8.35 | 35.22 | 102.83 | -0.058 | Chr.2 |
| Prupe.2G117200 | 930 | 106305.49 | 8.18 | 40.94 | 101.66 | -0.146 | Chr.2 |
| Prupe.2G117500 | 772 | 88948.59 | 8.62 | 43 | 103.6 | -0.185 | Chr.2 |
| Prupe.2G118000 | 766 | 87857.22 | 8.69 | 43.76 | 100.22 | -0.166 | Chr.2 |
| Prupe.2G121000 | 1149 | 129803.87 | 6.39 | 41.8 | 99.96 | -0.08 | Chr.2 |
| Prupe.2G126100 | 1172 | 132330.99 | 6.43 | 38.8 | 103.58 | -0.097 | Chr.2 |
| Prupe.2G126300 | 1074 | 121348.05 | 6.81 | 42.2 | 101.24 | -0.131 | Chr.2 |
| Prupe.2G126400 | 1176 | 133236.28 | 6.28 | 45.07 | 98.51 | -0.201 | Chr.2 |
| Prupe.2G126600 | 1023 | 116443.95 | 6.08 | 44.33 | 102.18 | -0.167 | Chr.2 |
| Prupe.2G126700 | 1147 | 129300.95 | 6.12 | 42.11 | 99.29 | -0.113 | Chr.2 |
| Prupe.2G126900 | 1176 | 133614.28 | 7.98 | 47.87 | 97.08 | -0.19 | Chr.2 |
| Prupe.2G127000 | 969 | 110095.07 | 6.71 | 39.09 | 99.04 | -0.136 | Chr.2 |
| Prupe.2G135700 | 1010 | 113754.47 | 6.54 | 41.64 | 100.78 | -0.109 | Chr.2 |
| Prupe.2G274900 | 882 | 101055.36 | 8.85 | 45.68 | 105.05 | -0.175 | Chr.2 |
| Prupe.2G283200 | 707 | 80675.76 | 6.66 | 49.42 | 104.63 | -0.146 | Chr.2 |
| Prupe.2G283300 | 933 | 107323.06 | 7.06 | 45.82 | 97.85 | -0.248 | Chr.2 |
| Prupe.2G283400 | 815 | 93641.38 | 7.84 | 44.66 | 100.44 | -0.245 | Chr.2 |
| Prupe.3G009700 | 1175 | 133193.99 | 7.03 | 42.18 | 94.97 | -0.254 | Chr.3 |
| Prupe.3G010100 | 1113 | 127002.05 | 7.56 | 42.41 | 95.26 | -0.252 | Chr.3 |
| Prupe.3G016700 | 1521 | 172191.93 | 5.48 | 41.48 | 102.26 | -0.143 | Chr.3 |
| Prupe.3G040600 | 1275 | 147530.85 | 8.35 | 53.71 | 98.78 | -0.305 | Chr.3 |
| Prupe.3G040800 | 1075 | 122943.24 | 9.61 | 42.35 | 95.83 | -0.381 | Chr.3 |
| Prupe.3G041000 | 1173 | 132532.48 | 9.28 | 44.74 | 102.57 | -0.253 | Chr.3 |
| Prupe.3G041100 | 615 | 70466.49 | 8.89 | 43.96 | 95.43 | -0.364 | Chr.3 |
| Prupe.3G041500 | 1359 | 156363.48 | 6.68 | 42.8 | 96.47 | -0.331 | Chr.3 |
| Prupe.3G054800 | 872 | 99027.67 | 5.69 | 46.84 | 99.14 | -0.128 | Chr.3 |
| Prupe.3G080600 | 880 | 99770.97 | 6.41 | 45.25 | 101.95 | -0.065 | Chr.3 |
| Prupe.3G082400 | 1136 | 129886.38 | 6.38 | 42.14 | 101.66 | -0.241 | Chr.3 |
| Prupe.3G083200 | 881 | 99928.07 | 5.93 | 42.98 | 103.6 | -0.047 | Chr.3 |
| Prupe.3G085000 | 1005 | 114509.67 | 6.06 | 39.93 | 101.8 | -0.208 | Chr.3 |
| Prupe.3G086700 | 786 | 90377.92 | 7.48 | 42.72 | 100.81 | -0.234 | Chr.3 |
| Prupe.3G087900 | 1317 | 150836.3 | 6.01 | 44.36 | 98.82 | -0.173 | Chr.3 |
| Prupe.3G088000 | 804 | 91403.82 | 6.67 | 38.55 | 97.55 | -0.261 | Chr.3 |
| Prupe.3G089000 | 761 | 87205.3 | 6.56 | 37.9 | 101.52 | -0.22 | Chr.3 |
| Prupe.3G089200 | 1030 | 117497.09 | 6.14 | 38.66 | 98.18 | -0.204 | Chr.3 |
| Prupe.3G089400 | 886 | 101268.12 | 5.86 | 40.5 | 97.75 | -0.206 | Chr.3 |
| Prupe.3G092700 | 994 | 113510.67 | 5.62 | 48.98 | 97.06 | -0.305 | Chr.3 |
| Prupe.3G100000 | 955 | 109121.12 | 8.44 | 47.27 | 101.55 | -0.134 | Chr.3 |
| Prupe.3G130900 | 985 | 112371.04 | 9 | 42.47 | 96.19 | -0.22 | Chr.3 |
| Prupe.3G141500 | 1368 | 154213.33 | 5.74 | 44.95 | 101.66 | -0.135 | Chr.3 |
| Prupe.4G038500 | 1029 | 116602.26 | 6.48 | 39.83 | 94.65 | -0.299 | Chr.4 |
| Prupe.4G109400 | 1166 | 133280.85 | 8.92 | 48.13 | 105.4 | -0.167 | Chr.4 |
| Prupe.4G149700 | 882 | 100233.04 | 8.83 | 43.93 | 98.32 | -0.296 | Chr.4 |
| Prupe.4G149800 | 891 | 101576.23 | 7.84 | 41.62 | 99.83 | -0.284 | Chr.4 |
| Prupe.4G149900 | 683 | 78186.28 | 8.95 | 44.49 | 93.97 | -0.381 | Chr.4 |
| Prupe.4G201800 | 973 | 110230 | 7.83 | 42.32 | 103.4 | -0.195 | Chr.4 |
| Prupe.4G224500 | 874 | 99893.38 | 6.2 | 42.96 | 96.04 | -0.243 | Chr.4 |
| Prupe.4G236500 | 421 | 48041.58 | 5.25 | 57.86 | 113.37 | -0.15 | Chr.4 |
| Prupe.4G260900 | 1008 | 114416.16 | 5.95 | 45.44 | 96.05 | -0.209 | Chr.4 |
| Prupe.4G284000 | 1028 | 118088.55 | 7.09 | 37.56 | 95.77 | -0.316 | Chr.4 |
| Prupe.4G284300 | 1026 | 118058.42 | 6.47 | 38.55 | 96.8 | -0.305 | Chr.4 |
| Prupe.4G284800 | 943 | 108451.18 | 6.27 | 37.14 | 95.81 | -0.309 | Chr.4 |
| Prupe.4G285100 | 699 | 79802.8 | 5.79 | 32.93 | 95.78 | -0.366 | Chr.4 |
| Prupe.4G285500 | 961 | 110090.21 | 6.14 | 36.81 | 95.53 | -0.269 | Chr.4 |
| Prupe.4G286000 | 1250 | 142084.46 | 6.34 | 44.28 | 100.66 | -0.186 | Chr.4 |
| Prupe.5G019000 | 1107 | 125071.19 | 5.26 | 39.06 | 97.62 | -0.172 | Chr.5 |
| Prupe.5G025000 | 944 | 108181.9 | 8.1 | 48.3 | 106.43 | -0.198 | Chr.5 |
| Prupe.5G025100 | 944 | 108017.56 | 7.78 | 49.78 | 105.82 | -0.198 | Chr.5 |
| Prupe.5G025200 | 947 | 108229.05 | 8.79 | 48.72 | 105.59 | -0.21 | Chr.5 |
| Prupe.5G025300 | 951 | 109238.74 | 9.33 | 37.98 | 102.68 | -0.203 | Chr.5 |
| Prupe.5G025400 | 949 | 108741.43 | 8.34 | 39.73 | 102.4 | -0.207 | Chr.5 |
| Prupe.5G025500 | 937 | 107820.46 | 8.71 | 39.11 | 102.64 | -0.205 | Chr.5 |
| Prupe.5G025600 | 952 | 109831.31 | 8.5 | 44.35 | 100.22 | -0.301 | Chr.5 |
| Prupe.5G111200 | 868 | 99376.67 | 8.72 | 36.63 | 104.21 | -0.175 | Chr.5 |
| Prupe.5G227800 | 874 | 99136.26 | 8.47 | 40.78 | 101.78 | -0.14 | Chr.5 |
| Prupe.6G083100 | 900 | 102841.33 | 8.66 | 42.53 | 96.56 | -0.178 | Chr.6 |
| Prupe.6G083200 | 1055 | 119890.59 | 7.25 | 43.95 | 94.64 | -0.129 | Chr.6 |
| Prupe.6G083400 | 1086 | 123964.77 | 6.64 | 46.72 | 94.15 | -0.201 | Chr.6 |
| Prupe.6G129800 | 1055 | 120291.39 | 5.99 | 49.99 | 106.17 | -0.173 | Chr.6 |
| Prupe.6G130000 | 1132 | 129645.08 | 6.01 | 53.01 | 101.09 | -0.201 | Chr.6 |
| Prupe.6G132100 | 920 | 105227.86 | 5.99 | 38.77 | 100.78 | -0.067 | Chr.6 |
| Prupe.6G146200 | 862 | 98786.16 | 7.5 | 51.94 | 102.97 | -0.122 | Chr.6 |
| Prupe.6G152300 | 1003 | 113695.9 | 5.73 | 42.71 | 97.85 | -0.201 | Chr.6 |
| Prupe.6G153300 | 621 | 71523.58 | 9.28 | 46.16 | 100.27 | -0.217 | Chr.6 |
| Prupe.6G153400 | 885 | 102080.94 | 8.57 | 49.73 | 104.06 | -0.215 | Chr.6 |
| Prupe.6G160100 | 1168 | 132644.96 | 7.72 | 38.06 | 95.79 | -0.226 | Chr.6 |
| Prupe.6G194900 | 899 | 102817.24 | 7.31 | 49.43 | 104.19 | -0.094 | Chr.6 |
| Prupe.6G199100 | 920 | 106296.67 | 7.25 | 53.49 | 98.65 | -0.134 | Chr.6 |
| Prupe.6G199400 | 912 | 104795.66 | 6.82 | 52.26 | 102.49 | -0.131 | Chr.6 |
| Prupe.6G199700 | 906 | 103665.71 | 6.39 | 48.79 | 102.53 | -0.156 | Chr.6 |
| Prupe.6G199800 | 905 | 103512.72 | 6.52 | 49.5 | 101.46 | -0.15 | Chr.6 |
| Prupe.6G200200 | 839 | 96043.86 | 6.31 | 47.75 | 101.55 | -0.155 | Chr.6 |
| Prupe.6G200400 | 742 | 85592.41 | 7.88 | 49.5 | 98.94 | -0.161 | Chr.6 |
| Prupe.6G200900 | 902 | 102685.78 | 6.39 | 49.43 | 100.3 | -0.142 | Chr.6 |
| Prupe.6G201100 | 907 | 103699.95 | 6.16 | 46.83 | 101.53 | -0.115 | Chr.6 |
| Prupe.6G243400 | 1216 | 138471.21 | 6.31 | 45.49 | 99.73 | -0.213 | Chr.6 |
| Prupe.6G243600 | 667 | 75519.35 | 9.05 | 40.64 | 98.05 | -0.278 | Chr.6 |
| Prupe.6G243700 | 1186 | 134292.13 | 8.02 | 41.04 | 100.13 | -0.151 | Chr.6 |
| Prupe.6G243800 | 1027 | 116807.43 | 6.91 | 42.44 | 99.97 | -0.233 | Chr.6 |
| Prupe.6G243900 | 1002 | 113548.02 | 6.54 | 47.42 | 101.81 | -0.114 | Chr.6 |
| Prupe.7G035000 | 1402 | 159906.51 | 6.14 | 47.54 | 97.86 | -0.196 | Chr.7 |
| Prupe.7G035600 | 1396 | 159443.56 | 6.3 | 46.2 | 101.34 | -0.177 | Chr.7 |
| Prupe.7G036900 | 847 | 97057.34 | 8.48 | 49.23 | 105.95 | -0.103 | Chr.7 |
| Prupe.7G037000 | 1132 | 129392.56 | 6.64 | 40.76 | 95.71 | -0.189 | Chr.7 |
| Prupe.7G037600 | 1048 | 119735.47 | 6.88 | 36.11 | 94.83 | -0.187 | Chr.7 |
| Prupe.7G062200 | 835 | 95253.62 | 6.23 | 43.62 | 97.22 | -0.229 | Chr.7 |
| Prupe.7G062500 | 830 | 94880.46 | 6 | 45.09 | 100.99 | -0.193 | Chr.7 |
| Prupe.7G063000 | 830 | 94811.34 | 7.55 | 42.56 | 99.95 | -0.238 | Chr.7 |
| Prupe.7G065300 | 852 | 96173.67 | 5.45 | 39.83 | 99.98 | -0.21 | Chr.7 |
| Prupe.7G065400 | 2026 | 230015.7 | 5.3 | 46.76 | 89.12 | -0.306 | Chr.7 |
| Prupe.7G065500 | 1889 | 212030.89 | 5.39 | 48.12 | 100.33 | -0.17 | Chr.7 |
| Prupe.7G138600 | 799 | 90196.28 | 6.03 | 43.03 | 108.21 | -0.047 | Chr.7 |
| Prupe.7G138700 | 784 | 88994.44 | 6.98 | 40.8 | 105.78 | -0.145 | Chr.7 |
| Prupe.7G138800 | 813 | 92701.91 | 6.71 | 37.37 | 103.46 | -0.147 | Chr.7 |
| Prupe.7G138900 | 816 | 91938.84 | 7.18 | 44.75 | 103.82 | -0.099 | Chr.7 |
| Prupe.7G139100 | 804 | 91255.55 | 8.1 | 45.41 | 104.27 | -0.102 | Chr.7 |
| Prupe.7G139200 | 812 | 92185.35 | 7.16 | 39.94 | 105.64 | -0.131 | Chr.7 |
| Prupe.7G139500 | 813 | 92573.97 | 7.72 | 39.53 | 105.63 | -0.141 | Chr.7 |
| Prupe.7G139600 | 820 | 93324.13 | 5.83 | 44.44 | 106.38 | -0.173 | Chr.7 |
| Prupe.7G139700 | 796 | 90935.91 | 6 | 41.95 | 103.67 | -0.237 | Chr.7 |
| Prupe.7G139800 | 794 | 90703.37 | 5.72 | 43.26 | 105.91 | -0.185 | Chr.7 |
| Prupe.8G005100 | 1166 | 132029.92 | 6.46 | 44.56 | 93.52 | -0.237 | Chr.8 |
| Prupe.8G005200 | 1128 | 127983.65 | 8.43 | 45.85 | 98.99 | -0.173 | Chr.8 |
| Prupe.8G005300 | 819 | 93122.46 | 8.63 | 39.81 | 107.17 | -0.062 | Chr.8 |
| Prupe.8G005400 | 1666 | 188586.37 | 6.12 | 45.99 | 92.88 | -0.174 | Chr.8 |
| Prupe.8G005500 | 1231 | 139335.02 | 6.44 | 43.1 | 98.77 | -0.104 | Chr.8 |
| Prupe.8G005600 | 1210 | 137536.04 | 6.18 | 44.54 | 93.16 | -0.145 | Chr.8 |
| Prupe.8G007600 | 914 | 104507.12 | 6.47 | 44.44 | 103.84 | -0.167 | Chr.8 |
| Prupe.8G008700 | 917 | 104958.99 | 8.3 | 54.49 | 100.31 | -0.185 | Chr.8 |
| Prupe.8G008900 | 899 | 102883.01 | 7.31 | 51.4 | 105.83 | -0.073 | Chr.8 |
| Prupe.8G009000 | 917 | 104950.19 | 8.45 | 52.74 | 100.62 | -0.184 | Chr.8 |
| Prupe.8G009300 | 779 | 89688.04 | 8.72 | 48.96 | 108.84 | -0.041 | Chr.8 |
| Prupe.8G009400 | 917 | 105243.59 | 8.57 | 52.36 | 97.22 | -0.212 | Chr.8 |
| Prupe.8G009600 | 917 | 105277.68 | 8.8 | 54.08 | 99.89 | -0.177 | Chr.8 |
| Prupe.8G009700 | 612 | 70111.73 | 8.09 | 59.26 | 103.35 | -0.12 | Chr.8 |
| Prupe.8G010000 | 896 | 103388.02 | 8.94 | 42.94 | 105.62 | -0.128 | Chr.8 |
| Prupe.8G010200 | 917 | 105304.81 | 8.65 | 42.64 | 105.86 | -0.109 | Chr.8 |
| Prupe.8G010600 | 949 | 108869 | 7.4 | 45.8 | 99.14 | -0.17 | Chr.8 |
| Prupe.8G010700 | 949 | 108265.86 | 5.78 | 45.94 | 102.07 | -0.132 | Chr.8 |
| Prupe.8G011900 | 999 | 114550.95 | 6.45 | 46.56 | 94.8 | -0.317 | Chr.8 |
| Prupe.8G012000 | 1046 | 118096.36 | 6.3 | 48.85 | 105.07 | -0.064 | Chr.8 |
| Prupe.8G012100 | 1054 | 119479.95 | 5.83 | 42.04 | 109.24 | 0.01 | Chr.8 |
| Prupe.8G021700 | 1189 | 134456.4 | 5.58 | 41 | 95.68 | -0.192 | Chr.8 |
| Prupe.8G022800 | 1188 | 135505.3 | 5.84 | 45.41 | 95.41 | -0.173 | Chr.8 |
| Prupe.8G023100 | 1162 | 132804.47 | 5.92 | 42.31 | 96.96 | -0.243 | Chr.8 |
| Prupe.8G023200 | 1182 | 135619.45 | 7.2 | 43.95 | 97.8 | -0.203 | Chr.8 |
| Prupe.8G023500 | 1190 | 136156.92 | 6.62 | 40.73 | 96.46 | -0.238 | Chr.8 |
| Prupe.8G023700 | 849 | 98132.6 | 7.61 | 45.49 | 101.26 | -0.128 | Chr.8 |
| Prupe.8G023800 | 1178 | 134516.95 | 5.94 | 46.75 | 100.12 | -0.199 | Chr.8 |
| Prupe.8G023900 | 1165 | 132829.01 | 7.89 | 47.38 | 95.73 | -0.212 | Chr.8 |
| Prupe.8G026500 | 1200 | 137714.42 | 7.82 | 39.03 | 90.38 | -0.261 | Chr.8 |
| Prupe.8G026700 | 1164 | 132950.54 | 7.02 | 41.45 | 91.01 | -0.268 | Chr.8 |
| Prupe.8G026800 | 1211 | 138670.79 | 7 | 41.31 | 93.03 | -0.233 | Chr.8 |
| Prupe.8G027000 | 818 | 93942.23 | 8.33 | 43.25 | 96.86 | -0.16 | Chr.8 |
| Prupe.8G027100 | 916 | 105391.43 | 6.4 | 41.07 | 89.61 | -0.347 | Chr.8 |
| Prupe.8G027300 | 1001 | 113948.16 | 6.31 | 42.92 | 97.18 | -0.173 | Chr.8 |
| Prupe.8G031800 | 1049 | 119059.11 | 6.39 | 39.97 | 101.42 | -0.086 | Chr.8 |
| Prupe.8G032100 | 901 | 101453.35 | 6.21 | 36.9 | 98.44 | -0.113 | Chr.8 |
| Prupe.8G032400 | 1200 | 135757.77 | 6.95 | 38.39 | 94.87 | -0.194 | Chr.8 |
| Prupe.8G034500 | 944 | 107066.19 | 7.4 | 37.3 | 102.01 | -0.125 | Chr.8 |
| Prupe.8G034900 | 834 | 94952.64 | 8.09 | 43.5 | 95.59 | -0.264 | Chr.8 |
| Prupe.8G037400 | 1217 | 140054.71 | 8.16 | 49.93 | 93.34 | -0.486 | Chr.8 |
| Prupe.8G037700 | 1179 | 135394.31 | 9.36 | 47.41 | 98.15 | -0.273 | Chr.8 |
| Prupe.8G041000 | 1241 | 141224.64 | 8.39 | 43.1 | 96.99 | -0.125 | Chr.8 |
| Prupe.8G044300 | 1254 | 142978.6 | 7.98 | 43 | 97.76 | -0.144 | Chr.8 |
| Prupe.8G044400 | 1262 | 143256.57 | 8.18 | 39.02 | 94.66 | -0.177 | Chr.8 |
| Prupe.8G046500 | 1104 | 125145.43 | 6.54 | 45.13 | 95.34 | -0.222 | Chr.8 |
| Prupe.8G046700 | 1304 | 147604.09 | 8.63 | 46.72 | 95.01 | -0.168 | Chr.8 |
| Prupe.8G053300 | 1234 | 140184.22 | 6.29 | 42.77 | 97.46 | -0.157 | Chr.8 |
| Prupe.8G056700 | 1108 | 126928.53 | 7.31 | 41.74 | 95.18 | -0.197 | Chr.8 |
| Prupe.8G077100 | 1175 | 132368.93 | 5.83 | 55.6 | 101.01 | -0.105 | Chr.8 |
| Prupe.8G077200 | 1205 | 134967.76 | 6.11 | 53.13 | 99.15 | -0.067 | Chr.8 |
| Prupe.8G077500 | 1236 | 139839.76 | 6.16 | 53.25 | 102.41 | -0.127 | Chr.8 |
| Prupe.8G077600 | 1172 | 130971.86 | 5.81 | 48.86 | 98.85 | -0.116 | Chr.8 |
| Prupe.8G102900 | 796 | 91286.16 | 5.44 | 47.01 | 101.39 | -0.199 | Chr.8 |
| Prupe.8G108500 | 1086 | 124002.86 | 8.42 | 39.69 | 99.22 | -0.145 | Chr.8 |
| Prupe.8G108800 | 913 | 103904.14 | 8.65 | 38.78 | 102 | -0.096 | Chr.8 |
| Prupe.8G109000 | 1104 | 125950.01 | 8.62 | 42.54 | 99.16 | -0.165 | Chr.8 |
| Prupe.8G109200 | 1169 | 133765.09 | 8.79 | 40.14 | 98.18 | -0.132 | Chr.8 |
| Prupe.8G109500 | 1080 | 123321.46 | 8.77 | 42.18 | 102.82 | -0.116 | Chr.8 |
| Prupe.8G110600 | 1165 | 132504.31 | 6.98 | 45.14 | 99.38 | -0.138 | Chr.8 |
| Prupe.8G110800 | 954 | 107891.55 | 5.14 | 40.48 | 100.28 | -0.15 | Chr.8 |
| Prupe.8G111200 | 1167 | 133002.08 | 6.17 | 39.62 | 94.41 | -0.275 | Chr.8 |
| Prupe.8G111500 | 1174 | 133619.95 | 5.41 | 42.76 | 94.02 | -0.292 | Chr.8 |
| Prupe.8G112100 | 1171 | 133732.43 | 5.7 | 43.02 | 92.85 | -0.292 | Chr.8 |
| Prupe.8G117900 | 1394 | 158463.92 | 6.11 | 48.79 | 105.22 | -0.122 | Chr.8 |
| Prupe.8G118900 | 953 | 108474.69 | 5.65 | 44.6 | 91.28 | -0.141 | Chr.8 |
| Prupe.8G127100 | 864 | 99128.12 | 9.23 | 52.5 | 103.23 | -0.266 | Chr.8 |
| Prupe.8G127300 | 838 | 95753.29 | 8.77 | 50.19 | 103.88 | -0.177 | Chr.8 |
| Prupe.8G132100 | 917 | 104293.08 | 8.85 | 37.12 | 101.21 | -0.134 | Chr.8 |
| Prupe.8G132300 | 866 | 98790.03 | 8.47 | 46.45 | 100.38 | -0.187 | Chr.8 |
| Prupe.8G132500 | 933 | 106345.8 | 8.9 | 47.4 | 99.34 | -0.25 | Chr.8 |
| Prupe.8G176400 | 1199 | 136477.25 | 6.08 | 46.23 | 91.38 | -0.293 | Chr.8 |
| Prupe.8G179400 | 1189 | 135743.56 | 6.38 | 48.67 | 91.65 | -0.308 | Chr.8 |
| Prupe.8G179500 | 1271 | 144467.14 | 5.66 | 45.28 | 93.48 | -0.258 | Chr.8 |
| Prupe.8G184900 | 1221 | 138453.29 | 7.88 | 49.49 | 95.68 | -0.197 | Chr.8 |
| Prupe.8G185200 | 1012 | 115198.48 | 8.33 | 42.78 | 93.27 | -0.271 | Chr.8 |
| Prupe.8G185300 | 1223 | 139249.67 | 6.9 | 43.47 | 92.13 | -0.253 | Chr.8 |
| Prupe.8G237200 | 1269 | 144309.58 | 6.15 | 47.5 | 94.71 | -0.254 | Chr.8 |

**Table S2.** The average number of CDS and UTR in subfamilies.

| **Genes** | **Average quantity of CDS** | **Average quantity of UTR** |
| --- | --- | --- |
| Subfamily Ⅰ | 3.31 | 5.80 |
| Subfamily Ⅱ | 6.16 | 4.19 |
| Subfamily Ⅲ | 5.72 | 1.55 |
| Subfamily Ⅳ | 4.22 | 1.63 |
| All | 4.69 | 4.47 |

**Table S3.** Details of primers used in the study.

| Gene ID | Primer Sequence |
| --- | --- |
| Prupe.1G217900 | TGCCTAGCCTCCACCGAT |
|  | TCCTCCGACCTGAAGCCT |
| Prupe.1G389500 | CCTCGAGCTGGCCAACAA |
|  | TCTCCATCTTCCGGGCCA |
| Prupe.1G545200 | GAACGGTTGGCCTCGACA |
|  | GCCTCCGTCACTTGCCTT |
| Prupe.2G022500 | GCGTCGGGAGGTGTTCAA |
|  | AACCGCCCCAATTGCAGA |
| Prupe.2G055200 | GTGGAAGGACCCATGCGG |
|  | CTTGTCATCGGCCCTGCA |
| Prupe.2G060400 | AAAACGGGCGGGTTTTGC |
|  | TTGTTCCGCCCAGAGCTG |
| Prupe.2G283200 | AGGGGCATGGAGTCGAGT |
|  | GGCATCAGAGGAACTGCTGT |
| Prupe.2G283300 | TCTGCCAGGGATGAGCTGA |
|  | GGTCTTGCCAAGTCCTCCC |
| Prupe.3G016700 | GTGCCACGCCCTCTCAAA |
|  | GGCGAGTGTGGTCTTCCC |
| Prupe.4G201800 | TGTGCAGTGCTAGTGCCC |
|  | TGGCAGGCCACATGAACT |
| Prupe.4G224500 | ACGCAACAGGTCATGGCA |
|  | AGCGAACGGGTCAGCATC |
| Prupe.4G284000 | GAACGGTTGGCCTCGACA |
|  | ACTTGGCGTTGCTCTGCA |
| Prupe.5G019000 | AGCTTCGGGGATGCACTG |
|  | GACAGAGTGAACCCCGCC |
| Prupe.5G025600 | GGGAAGACCACCTTGGCC |
|  | ACAGTGATCCACGCACGG |
| Prupe.6G083400 | GGCATTGGAGGGAGTCGG |
|  | CGCAGCCTACTTCGTGGT |
| Prupe.6G243400 | TTGCAGCATCCCTCACCC |
|  | TTCGGCCAACCCCACAAG |
| Prupe.7G065500 | GGGGGCCTTCCGCATATC |
|  | GAGCGGCGCTTTGTTCAC |
| Prupe.7G138600 | CGGGGTTCGATGTGCAGT |
|  | GTTTTCCCGCATCCCCCA |
| Prupe.7G139100 | TGCAGCATCGGTCAAGCT |
|  | TCACAGAGCTTGGCAGGC |
| Prupe.8G023100 | GAGCGAAGGAGCCAACGT |
|  | TGTGTGTACCTCAGCGGC |
| Prupe.8G023800 | GCAGACACTCGCAGGGTT |
|  | CGGTCAACAGCTCCGGAG |
| Prupe.8G027300 | GCGGCACACTGGAATCCT |
|  | TGGCAGGCATTTGGAGGG |
| Prupe.6G152300::GFP | ggacagggtacccggggatccATGGCATCAGCTGGCGTAGA |
|  | caccatggtactagtgtcgacAAGAAATAATAAGACCTAAAAAGATCTTCAA |
| Prupe.2G274900::GFP | ggacagggtacccggggatccATGGCTCCTATGGCCAACCA |
|  | caccatggtactagtgtcgacTTAGATTCAAGTACTTCAAATTTGGGA |
| Prupe.7G138800::GFP | ggacagggtacccggggatccATGGCAGTGGATTTAGTCGGAG |
|  | caccatggtactagtgtcgacTCAAGATCGAAGCTTGTGGAGC |
